# The genomic analysis of current-day North African populations reveals the existence of trans-Saharan migrations with different origins and dates

**DOI:** 10.1007/s00439-022-02503-3

**Published:** 2022-11-28

**Authors:** Marcel Lucas-Sánchez, Karima Fadhlaoui-Zid, David Comas

**Affiliations:** 1 Institut de Biologia Evolutiva (CSIC-Universitat Pompeu Fabra), Departament de Medicina i Ciències de la Vida, Universitat Pompeu Fabra, Barcelona, Spain; 2 Laboratory of Genetics, Immunology, and Human Pathologies, Faculty of Science of Tunis, University of Tunis El Manar, Tunis, Tunisia; 3 College of Science, Department of Biology, Taibah University, Al Madinah Al Monawarah, Saudi Arabia

## Abstract

The Sahara Desert has acted as a barrier to human gene-flow between the northern and central parts of Africa since its aridification. Nonetheless, some contacts between both sides of the desert have occurred throughout history, mainly driven by commercial activity. Part of this was the infamous trans-Saharan slave trade, which forcedly brought peoples from south of the Sahara to North Africa from Roman times until the nineteenth century. Although historical records exist, the genetic aspects of these trans-Saharan migrations have not been deeply studied. In the present study, we assess the genetic influence of trans-Saharan migrations in current-day North Africa and characterize its amount, geographical origin, and dates. We confirm the heterogeneous and generally low-frequency presence of genomic segments of sub-Saharan origin in present-day North Africans acquired in recent historical times, and we show evidence of at least two admixture events: one dated around the thirteenth–fourteenth centuries CE between North Africans and a Western-sub-Saharan-like source similar to current-day Senegambian populations, and another one dated around the seventeenth century CE involving Tunisians and an Eastern-sub-Saharan-like source related to current-day south-Sudan and Kenyan populations. Time and location coincide with the peak of trans-Saharan slave-trade activity between Western African empires and North African powers, and are also concordant with the possibility of continuous recent south-to-north gene-flow. These findings confirm the trans-Saharan human genetic contacts, providing new and precise evidence about its possible dates and geographical origins, which are pivotal to understanding the genomic composition of an underrepresented region such as North Africa.

**Supplementary Information:**

The online version contains supplementary material available at 10.1007/s00439-022-02503-3.

## Introduction

North African peoples have received genetic contributions from different surrounding populations throughout history, and ancestry studies have identified at least four main genetic components in their genomes (Henn et al. 2012; Arauna et al. 2017; Serra-Vidal et al. 2019; Lucas-Sánchez et al. 2021a), found at different proportions depending on the population: an autochthonous North African component that follows a west-to-east gradient, a Middle Eastern-like component in an east-to-west gradient, an European-like component, and a sub-Saharan-African-like component. These components have been linked to documented historical events, like the Neolithic expansion (~ 7500–5000 years ago) and the Arab conquest coming from the Middle East (seventh–eleventh centuries CE), the arrival of historic Mediterranean powers like the Greeks, the Romans, or the Ottomans, and the long-lasting trans-Saharan slave-trade (from Roman times to the nineteenth century CE) (Hiernaux 1975; McEvedy 1995; Newman 1995; Lucas-Sánchez et al. 2021a). However, in general terms, North Africa has been usually neglected from genetic studies, with a worrying scarcity of data when compared to other world regions, which leaves many questions unanswered or poorly understood.

One of the less-addressed questions is the characterization of the genetic influence of trans-Saharan migrations in North African populations. The African continent contains the largest amount of human genetic diversity of any world regions, as it was the place of origin of the *Homo sapiens* species (Tishkoff et al. 2009; Scheinfeldt et al. 2010) and its populations have been diversifying since then, while all non-African populations come from an out-of-Africa bottleneck, which reduced their genetic diversity (Rosenberg et al. 2002; Ramachandran et al. 2005; Tishkoff et al. 2009). But inside Africa, there is a major barrier to contacts and gene-flow between populations, the Sahara Desert, which has acted as genetic barrier separating populations at both sides of the desert. Still, this barrier has not been totally impermeable. Contacts between peoples north and south of the Sahara have occurred since prehistoric times, when the climate conditions were warmer and wetter, thus facilitating trans-Saharan migrations (Brooks et al. 2005), all throughout history, with commercial contacts between northern and southern states and, more importantly, the infamous trading of slaves from south of the Sahara that lasted from the Roman presence in North Africa (first century BCE) until very recently in the nineteenth century CE (Newman 1995; Segal 2002; Harich et al. 2010; Fadhlaoui-Zid et al. 2011; Pellat et al. 2012; Arauna et al. 2017; Lucas-Sánchez et al. 2021a).

Trading through the Sahara started in prehistory but was especially relevant from the eighth to the seventeenth centuries CE (Shillington 2022; Bovill 1968; Newman 1995; Falola and Heaton 2008). Trading routes also involved cultural exchange, which led to the expansion of Islam south of the Sahara. In Western Africa several routes were established, powered by the introduction of Muslim camel caravans (Newman 1995; Wright 2007; Harich et al. 2010), connecting with current-day Morocco and, with lesser importance, Algeria and Tunisia (Lovejoy 1983; Lewicki 1992; Newman 1995; Masonen et al. 1997; Segal 2002; Harich et al. 2010). South of the desert, these routes arrived mainly at the Ghana Empire (third–thirteenth centuries CE)—in current-day Mali, Senegal and southern Mauritania-, and the Mali Empire (thirteenth–seventeenth centuries CE)—which expanded the Ghana Empire territory to current-day Gambia, Guinea-Bissau, Côte d'Ivoire, northern Ghana and Burkina Faso, and eastern Niger (Segal 2002; Falola and Heaton 2008; Messier and Miller 2015). Through these routes, salt and gold were the main trading goods, but also an important number of people were brought as slaves from Western Africa to the north. It is roughly estimated that through all the years of trans-Saharan slave trading, around 5 million people (Lovejoy 1983; Harich et al. 2010), and even maybe up to 9 million according to some authors (Inikori 1982); Austen 1987; Elikia Mbokolo 1995), were taken by force from Western to Northern Africa. Some authors state that trans-Saharan slave trade involved as many people as the far more studied Atlantic slave trade, while it expanded much longer in time (Segal 2002; Harich et al. 2010).

Previous genetic studies have found substantial variation in the amount of sub-Saharan-like component in different North African populations (Idaghdour et al. 2010; Henn et al. 2012; Arauna and Comas 2017; Serra-Vidal et al. 2019; Lucas-Sánchez et al. 2021b), being relatively low in the majority of samples and with the highest proportions found in some individuals from southern Morocco (Henn et al. 2012; Arauna et al. 2017; Serra-Vidal et al. 2019), some Algerian Imazighen groups (Arauna et al. 2017; Serra-Vidal et al. 2019), some Arab Tunisian individuals (Lucas-Sánchez et al. 2021b), and some Saharawi (Serra-Vidal et al. 2019). This variability in the proportion of sub-Saharan-like component is suggested to be linked to social structure related to slave trade (Harich et al. 2010; Fadhlaoui-Zid et al. 2011; Henn et al. 2012; Arauna et al. 2017). Previous estimated dates of trans-Saharan migrations to Northern Africa coincide with the periods of largest western trans-Saharan slave-trade activity (Henn et al. 2012; Arauna et al. 2017): the rise of the Ghana Empire and the “Great Berber Uprising”, which led to important slave trade from Western African empires to newly established kingdoms north of the Sahara (Newman 1995; Oliver and Atmore 2001; Henn et al. 2012), and to historically documented trade carried out by the Arabs and the Ottomans, who ruled in the region in different periods, and the Crown of Castille and the Portuguese Kingdom, who forcedly took West African people sometimes through North Africa to their newly stablished Atlantic colonies (Newman 1995; Oliver and Atmore 2001; Mosto 2003; Arauna et al. 2017). Specific dates differ among publications, covering the mentioned periods and spanning from the ninth (Henn et al. 2012) to the thirteenth, seventeenth, and nineteenth centuries CE (Arauna et al. 2017) depending on the samples studied. Nevertheless, they all agree with a recent slave-trade related admixture that has probably been continuous in the last few centuries. The recent estimation dates of these migrations are also supported by the large amount of variation of the sub-Saharan-like ancestry among the studied samples and the mitochondrial DNA (mtDNA) evidence, which shows a specific connection between haplogroups present in North Africa and the known trans-Saharan slave routes during the times of the Arab expansion (Harich et al. 2010). The spread of sub-Saharan genetic influence did not always stop in North Africa, as sub-Saharan-like ancestry coming from gene flow with North Africa has been described in European and Middle Eastern populations (Moorjani et al. 2011).

However, genomic population analyses that have explored the gene pool of North African groups did not focus on the analysis of the influence of trans-Saharan migrations. In the present study, we aim to assess the genetic influence of trans-Saharan migrations in North African populations through the analysis of new genome-wide data from Tunisian and Moroccan individuals applying novel state-of-the-art methods to identify, quantify, and date the sub-Saharan-like genetic component in the region, as well as to locate its possible geographical origin. We applied local ancestry-based methods to identify the most probable origin of the sub-Saharan-like sources involved in recent admixture events in North Africa and expand the findings of previous research. We chose new state-of-the-art methods that upgrade previous approaches and have not been used to investigate this question, and thus can provide new and more precise evidence. Our findings confirm the recent character of the sub-Saharan admixture in North Africa, with dates overlapping with the strongest times of trans-Saharan slave trade and provide more precise evidence on its possible geographical origins.

## Results

### Population structure

**Fig. 1:**
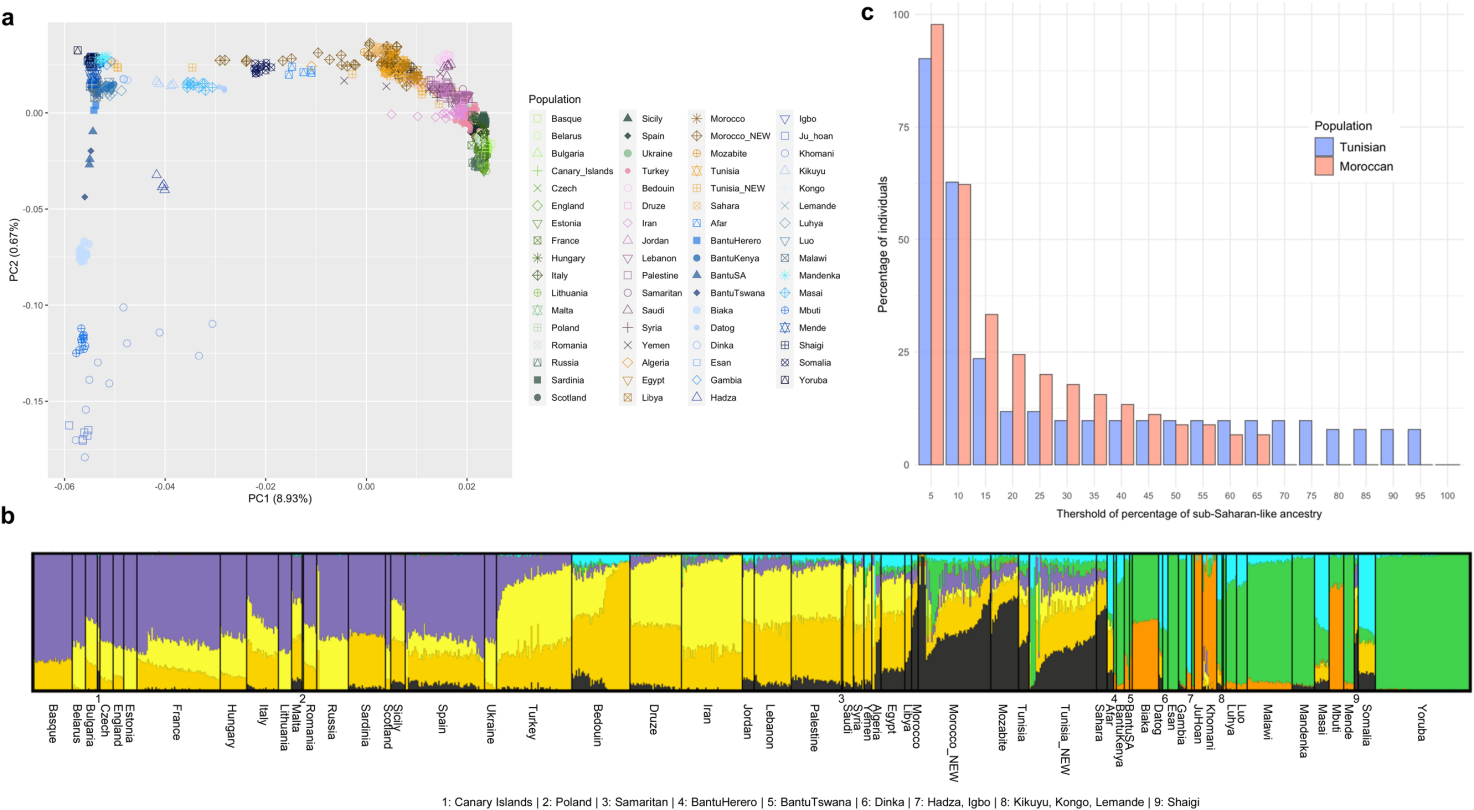
General population structure and percentage of sub-Saharan-like ancestry in North African samples. **a** Principal component analysis of the newly genotyped Tunisian and Moroccan samples (with the “NEW” label in the legend) along with a panel of reference populations from Europe (in green), the Middle East (in pink and purple), North Africa (in yellow and brown), and sub-Saharan Africa (in blue). **b** ADMIXTURE analysis for *K* = 7. **c** Percentage of sub-Saharan-like ancestry in newly genotyped Tunisian and Moroccan samples. Each bar represents the percentage of individuals (*y*-axis) with at least an amount of sub-Saharan-like ancestry specified by its position in the *x*-axis, separated by population. Ancestry percentages are inferred by the local ancestry inference algorithm RFMix v1.5.4 (Maples et al. 2013)

We obtained genome-wide data for 109 individuals from Tunisia and Morocco, 96 of which passed the relatedness filter (kinship coefficient < 0.0442), yielding a total of 425,777 SNPs (see “Materials and methods”). The projection of the 96 newly genotyped samples with a panel of relevant world-wide populations in a principal component space (Fig. 1a, Supplementary Fig. 1) shows the expected distribution of samples according to their geographical region of origin, with PC1 separating sub-Saharan African individuals from the rest of the populations and PC2 presenting a Europe-Middle East-North Africa cline. North African populations are placed in between the Middle Eastern and sub-Saharan African samples, with most samples being closer to the Middle Eastern ones. Nonetheless, some individuals from our newly genotyped data cluster with sub-Saharan African samples. An ADMIXTURE analysis performed with the same dataset (Fig. 1b, Supplementary Fig. 2) showed North Africans exhibiting different proportions of four components: one that is maximized in Europeans, one in Middle Easterns, one in North Africans themselves, and one in some sub-Saharan African populations. This last one, though, is presented, in general, in small proportions.

### Local ancestry inference

The first aim of our study was to identify sub-Saharan-like ancestry segments in North African populations and explore their proportions in North African genomes. Local ancestry inference (LAI) in both Tunisians and Moroccans reveals a similar pattern for the sub-Saharan-like component, which represents a low or medium–low percentage in most of the samples, although some heterogeneity is found, with samples that exhibit large amounts of such component (Fig. 1c, Supplementary Table 4).

### Geographical and temporal characterization of the sub-Saharan-like component

A main goal of our study was to determine the most probable origin of the source populations participating in the admixture from south of the Sahara. We approached this question with two main lines of local ancestry-based analyses: ancestry-specific principal component analysis (ASPCA) (Moreno-Estrada et al. 2013; Browning et al. 2016) and MOSAIC (Salter-Townshend and Myers 2019).

**Fig. 2:**
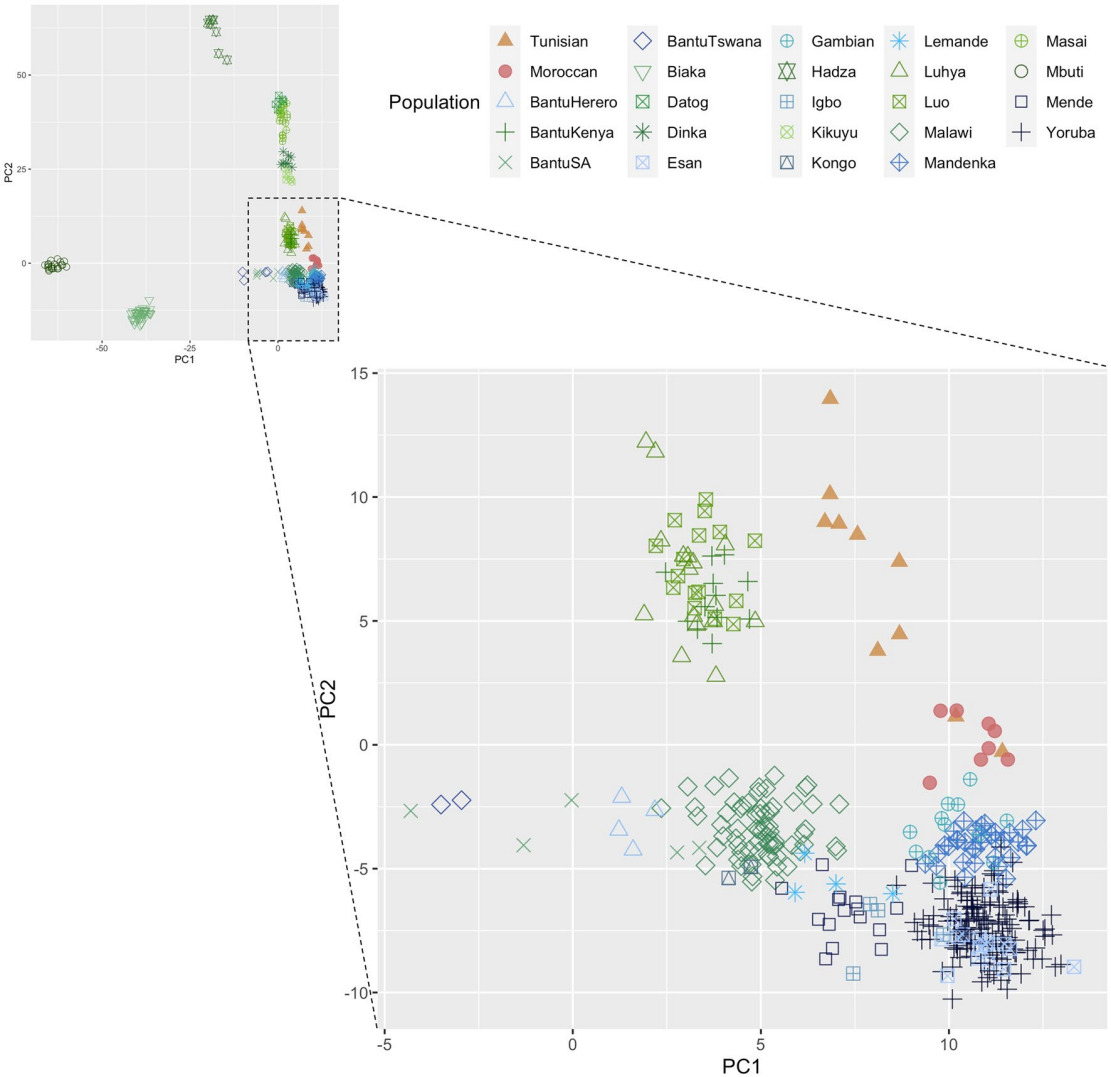
Ancestry-specific PCA for sub-Saharan-like ancestry at a 50% threshold. Plotted are North African haplotypes masked to keep only sites assigned to a sub-Saharan-like ancestry and whose sub-Saharan-like ancestry is inferred to be at least 50%, and reference sub-Saharan Africa populations’ haplotypes. These populations are presented in two color ranges: in green are haplotypes from eastern populations and in blue, from western populations. The bottom-right panel is a zoom-in of the designated region in the top-left panel

In the ASPCA, we plotted North African samples masked to retain only the sub-Saharan-like ancestry with current-day African individuals from south of the Sahara (Fig. 2). When the threshold of such ancestry is set at 50% (keeping only North Africans with high sub-Saharan-like ancestry), North African samples cluster closely, but exhibit a differentiation between a cluster of four Tunisians (8 haplotypes in the plot) which is closer to a cluster of current-day Kenyan individuals (Luhya, Luo, and Bantu from Kenya), and a cluster of four Moroccans and one Tunisian (10 haplotypes) which appears closer to a current-day Senegambian cluster (samples from Gambian and Mandenka populations). This differentiation was corroborated by checking the mean ASPCA Euclidean distance from each North African haplotype to each African population from south of the Sahara (Supplementary Table 1), and by comparing dot-to-dot ASPCA distances using *t* tests (Supplementary Note 1). These analyses confirmed that, in terms of ASPCA distance: (1) Tunisians (either including or excluding the sample closer to Senegambian populations) are significantly closer to current-day Kenyan populations than to current-day Senegambian populations (*p* < 2.2 × 10^–16^), (2) Moroccans are significantly closer to the later (*p* < 2.2 × 10^–16^), and (3) Moroccans and Tunisians significantly differ in their distance to each of these two sub-Saharan Africa clusters (*p* < 2.2 × 10^–16^ in both comparisons).

Lowering the threshold of sub-Saharan-like ancestry to 40% and 30% maintains this tendency, as new entering samples are Moroccans that cluster closer to Senegambian populations (Supplementary Fig. 3, top panels). When the threshold is set to 20%, North African samples start to mix, and with a 10% threshold, the differentiation between the two North African populations is completely lost (Supplementary Fig. 3, bottom panels).

**Fig. 3:**
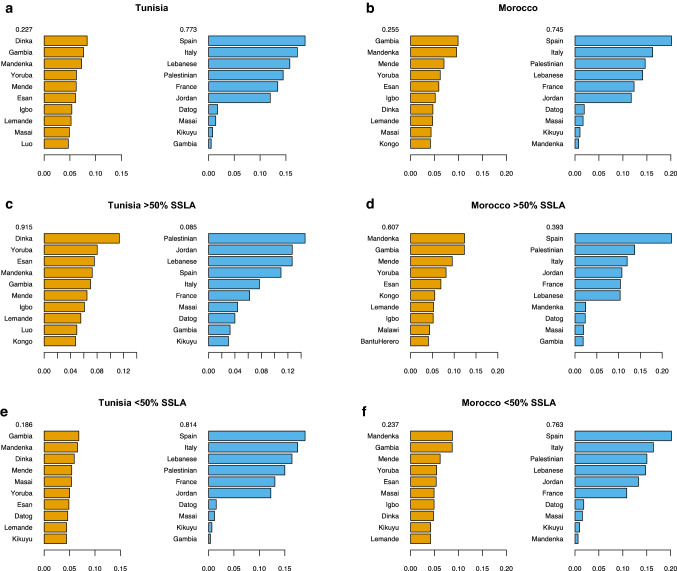
MOSAIC copying probabilities. Inferred copying probabilities for **a** the whole Tunisian population, **b** the whole Moroccan population, **c** Tunisian and **d** Moroccan individuals with > 50% of sub-Saharan-like ancestry (SSLA), and **e** Tunisian and **f** Moroccan individuals with < 50% of sub-Saharan-like ancestry. The ten populations (*y*-axes) with higher copying proportions (*x*-axes) are plotted for each of the two inferred sources of admixture. Numbers above each inferred source represent the proportion of the source in the admixed population ancestry profile. These ancestry proportions are estimated genome-wide and averaged across all admixed target individuals

**Fig. 4:**
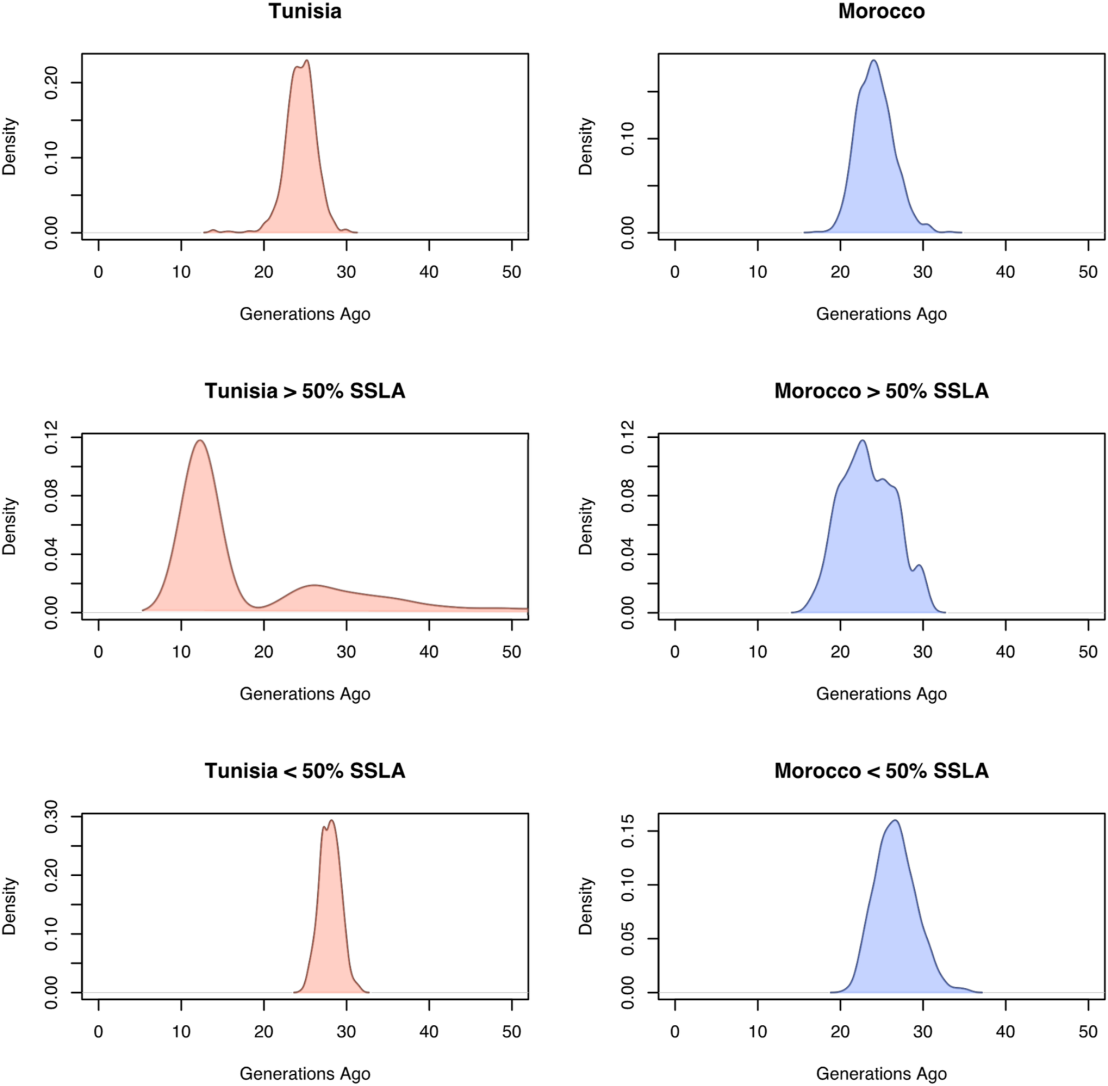
Admixture dates inferred by MOSAIC’s ancestry-aware coancestry curves and bootstrap analysis. Each panel shows the distribution of admixture dates in generations before present (*x*-axes) obtained by bootstrapping by individual the ancestry-aware coancestry curves inferred by MOSAIC. Admixture dates are presented for **a** the whole Tunisian population, **b** the whole Moroccan population, **c** Tunisian and **d** Moroccan individuals with > 50% of sub-Saharan-like ancestry (SSLA), and **e** Tunisian and **f** Moroccan individuals with < 50% of sub-Saharan-like ancestry

To further explore the origin location and composition of the ancestral admixing sub-Saharan groups as well as dating the admixture events, we applied the local-ancestry-based software MOSAIC (Salter-Townshend and Myers 2019). In Tunisian individuals, MOSAIC infers admixture between a major non-sub-Saharan-like source contributing to a 77% of the admixed population, and a minor sub-Saharan-like source with a contribution of 23% (Fig. 3a). Focusing on the sub-Saharan-like source, the current-day populations with higher copying probabilities are the Dinka, an eastern population, and then the Gambian and Mandenka, from the Senegambian region. These are followed, with lower probabilities, by Yoruba, Mende, and Esan, also western sub-Saharan African populations. This presence of eastern and western populations in the top positions of the copying probabilities matrix suggests different sources for the sub-Saharan-like component in the Tunisian population. The ancestry-aware coancestry curves reveal evidence of admixture between the two inferred sources dated to around 24–25 generations ago (Supplementary Fig. 4), in the thirteenth century CE, with accurate estimates in the bootstrap analysis (Fig. 4a). The curves of both the point and the bootstrap analyses hint again to the presence of two different genetic inputs in Tunisia from south of the Sahara. The curve that considers only the sub-Saharan-like source is dated much younger than the one considering the two admixing groups, around 18–19 generations ago (Supplementary Fig. 4). This is even clearer in the bootstrap analysis considering only the sub-Saharan-like source, in which we can see two peaks, one large peak around 15 generations ago (sixteenth century CE) and a smaller peak around 25 generations ago (Supplementary Fig. 5, left panel).

To test if this differentiation could be related to the different individual amounts of the sub-Saharan-like genetic component within the Tunisian population, we computed MOSAIC again in two subsets of individuals: one with the 46 Tunisians with < 50% inferred sub-Saharan-like ancestry, and one with the five Tunisians with > 50% of such ancestry. In Tunisians with > 50% of sub-Saharan-like ancestry, MOSAIC infers admixture with a major sub-Saharan-like source (91% of the admixture proportions) with the Dinka as the highest current-day population in the copying probabilities matrix, followed by a group of Nigerian and Senegambian populations in significantly lower proportions (Fig. 3c). These results are consistent with those of the ASPCA (Fig. 2), placing the Tunisians with highest sub-Saharan-like ancestry closer to current-day eastern sub-Saharan populations, suggesting an eastern origin for the admixture source. The inferred admixture date in the non-bootstrapped coancestry curve analysis is around 12 generations ago (Supplementary Fig. 6). The density plot of the bootstrap analysis shows a major peak also around 12 generations, with a smaller peak around 25 generations ago (Fig. 4c). In contrast, and in agreement with our previous results, in Tunisians with < 50% of sub-Saharan-like component an admixture dated around 28 generations ago (twelfth–thirteenth century CE) is inferred with a minor sub-Saharan-like source (19%) mostly copying from Gambian and Mandenka current-day populations (Figs. 3e, 4e and Supplementary Fig. 7).

The two admixture sources inferred in the Moroccan population are also a major non-sub-Saharan-like source (75%) and a minor sub-Saharan-like source (25%), with Gambian and Mandenka as the closest current-day populations in the copying probabilities matrix (Fig. 3b), coinciding with Tunisians with < 50% sub-Saharan-like component. The ancestry-aware coancestry curve and its bootstrap analysis date this admixture around 23–24 generations ago (late thirteenth–early fourteenth century CE), matching with the inferred admixture in Tunisians (Fig. 4b and Supplementary Fig. 8). When applying MOSAIC only to the four Moroccans with > 50% sub-Saharan-like component, the composition of the sub-Saharan-like admixture source remains the same, copying mainly from current-day Senegambian populations, but with a higher contribution (61%) to the admixed population (Fig. 3d). The inferred admixture dates are consistent with those in the whole Moroccan population with similar estimated dates, around 22–23 generations ago (Fig. 4d and Supplementary Fig. 9). No relevant differences are found in the copying probabilities matrix when comparing Moroccans with < 50% of sub-Saharan-like ancestry with the complete Moroccan dataset (Fig. 3f). Admixture in this subset is dated around 25–26 generations ago (Fig. 4f and Supplementary Fig. 10).

Since haplotype-based methods, such as MOSAIC, might be biased toward the detection of recent events, simulated data were produced to discard ancient trans-Saharan gene flow in the present samples (see Supplementary Note 2). The higher likelihood of a recent single-wave admixture over the existence of a recent and an older waves in North Africa was confirmed by comparing the sub-Saharan-like segment-length distribution in our populations with simulated one-wave and two-wave admixed populations with equivalent ancestral profiles (Supplementary Note 2, Supplementary Figs. 11 and 12, Supplementary Table 3), thus corroborating that the sub-Saharan-like genetic component in current North Africans is related to a recent admixture event and discarding main contributions from older trans-Saharan gene flow events.

To make sure that the two methods to assess local ancestry (RFMix v2 and MOSAIC) give concordant results regarding inferred local ancestry proportions, the sub-Saharan-like proportions inferred by each method were compared through all individuals in the dataset (Supplementary Fig. 13). The two methods present a high correlation (*r*^2^ > 0.95, *p* < 2.2 × 10^–16^) and there are no significant differences between the two groups of inferred sub-Saharan-like proportions (*p* < 0.01).

### Temporal trajectory of the sub-Saharan-like component

To further characterize the sub-Saharan-like component in North Africa, we explored its proportions relative to the total effective population size through time. Regarding total effective population size, both Tunisians and Moroccans exhibit similar trajectories, although Moroccans show evidence of a bottleneck followed by a population growth around 16 generations ago, which assuming 30 years per generation (Tremblay and Vézina 2000) would be dated around the sixteenth century CE (Supplementary Fig. 14).

**Fig. 5:**
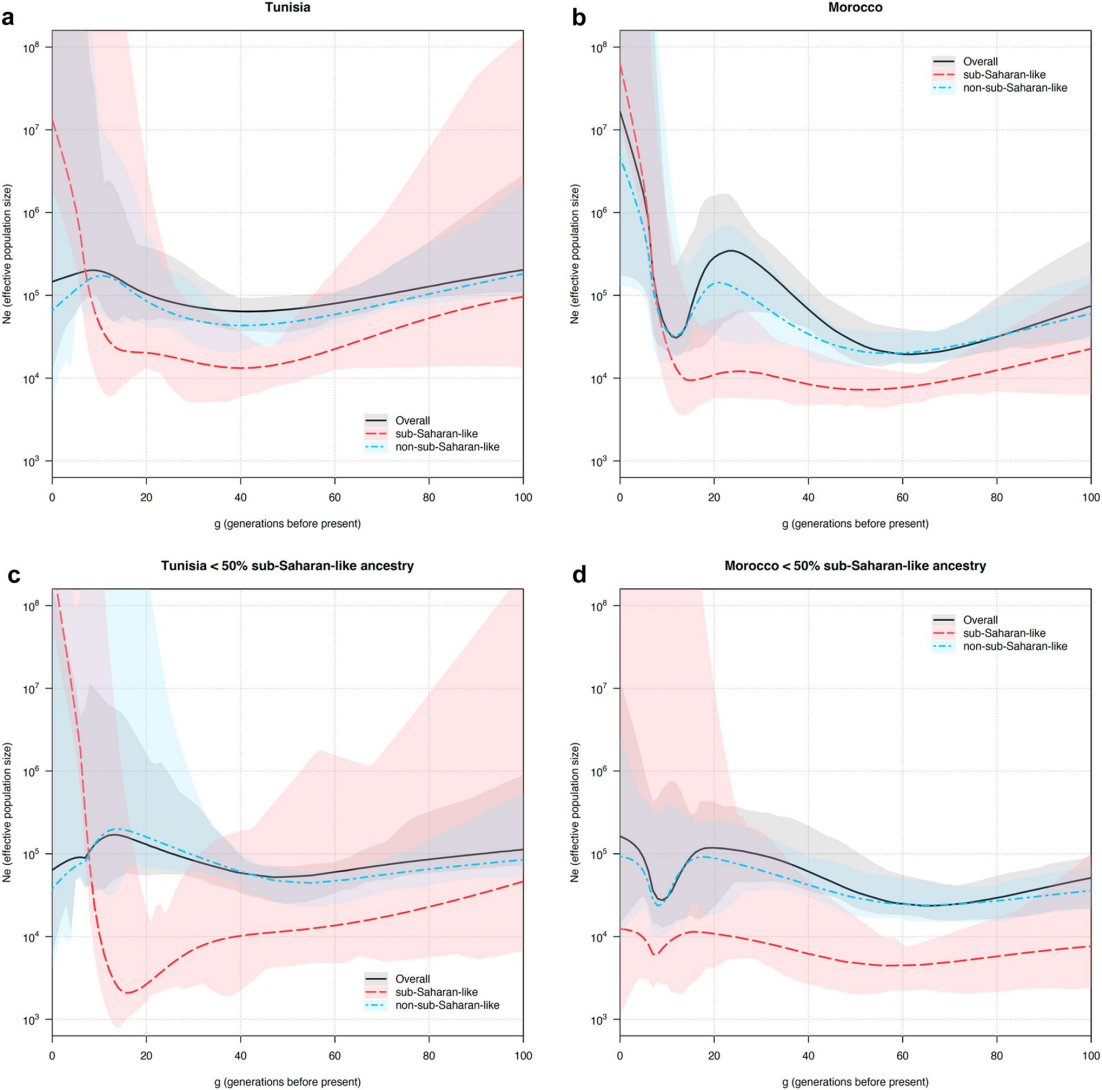
Estimated ancestry-specific effective population size. Effective population size (*N*_e_) is plotted on a log scale (*y*-axes) and divided in overall *N*_e_ (in black), *N*_e_ for the sub-Saharan-like ancestry (in red), and *N*_e_ for the non-sub-Saharan-like ancestry (in blue). The x-axes show generations before the present. **a** and **b** *N*_e_ in the whole Tunisian and Moroccan population, respectively. **c** and **d** *N*_e_ in Tunisian and Moroccan individuals, respectively, with < 50% of inferred sub-Saharan-like ancestry. Colored around the lines are the 95% bootstrap confidence intervals

We then divided IBD segments per ancestry (sub-Saharan-like versus non-sub-Saharan-like components) (Fig. 5) and observed that the non-sub-Saharan-like component follows a similar trajectory to the overall effective population size, which is expected considering the low proportions of the sub-Saharan-like component (Fig. 5a, b). The sub-Saharan-like component follows a similar pattern in Tunisians and Moroccans, with historically lower effective population sizes than the non-sub-Saharan-like component but with an increase starting around 17–18 generations ago in Tunisians (late fifteenth–early sixteenth century CE) and a bit later, around 16–17 generations in Moroccans (sixteenth century CE). This increase in population size lasts until present dates (Fig. 5a, b). In both cases, the increase in the effective population size of the sub-Saharan-like component coincides with a decrease in the non-sub-Saharan-like component, which can be an evidence of admixture (Browning et al. 2018). It is noteworthy that in Moroccans, after the initial decay of the non-sub-Saharan-like effective population size in the potential admixture, both ancestries increase together in effective population size.

To discard that this signal found in the sub-Saharan-like component was carried by sub-Saharan African individuals not involved in the admixture event, we computed the effective population size only in individuals from south of the Sahara (Supplementary Fig. 15). We do not see the increase in effective population size that we see in the sub-Saharan-like component carried by North African individuals, which agrees with this signal being related to the admixture event.

To avoid artifacts due to the presence of individuals with high proportions of sub-Saharan-like ancestry, we replicated the analysis excluding individuals with large sub-Saharan-like genetic component and focusing on those with < 50% inferred sub-Saharan-like ancestry. In Tunisians, after removing the five individuals with higher sub-Saharan-like ancestry, the sub-Saharan-like component follows a similar trajectory than in the whole Tunisian dataset, with just a slight increase in the strength of the observed bottleneck (Fig. 5c). In contrast, removing the four Moroccans with higher sub-Saharan-like ancestry changes the results found in the whole Moroccan dataset, as the sub-Saharan-like component now follows a similar trajectory than that of the non-sub-Saharan-like component (Fig. 5d). It is noteworthy that not only the sub-Saharan-like component changes after removing these four individuals, but the pattern of the non-sub-Saharan-like component in recent generations also changes from a population growth to a steady population size, maintaining the bottleneck previously observed around 16 generations ago. This result suggests that the growth in effective population size observed in the whole Moroccan dataset (Supplementary Fig. 14) is tied to those individuals with > 50% of sub-Saharan-like component rather than to the whole population.

## Discussion

Extant North African populations show an amalgam of ancestry components, and inferring local ancestry is challenging, since the Middle Eastern-like, the European-like, and the autochthonous ancestral genetic components might be difficult to accurately differentiate (Henn et al. 2012; Arauna et al. 2017; Lucas-Sánchez et al. 2021a). In contrast, the sub-Saharan-like component is relatively easier to identify because of its higher genetic differentiation from the out-of-Africa components (Henn et al. 2012; Arauna et al. 2017; Lucas-Sánchez et al. 2021a). However, populations south of the Sahara exhibit the highest inter-population diversity in any world region (Reed and Tishkoff 2006; Campbell and Tishkoff 2008; Tishkoff et al. 2009), and grouping them together as a single ancestry might be misleading. In the present study, we have assessed the sub-Saharan-like components inferred in North Africa in the wide diversity range of populations south of the Sahara to estimate the amount, origin, and tempo of the trans-Saharan gene flow into Northern Africa. For ease of reading, we have used the term “sub-Saharan” to refer to African populations south of the central latitudinal line of the Sahara Desert. We are aware that, as mentioned, these include a wide range of genetic and cultural diversity, and we use it with a solely geographical meaning due to the need to differentiate the two sides involved in admixture events related to trans-Saharan migrations while aiming to pinpoint its specific geographical origins.

We have been able to identify sub-Saharan-like ancestry segments in the genomes of individuals from Tunisia and Morocco, corroborating that there have been contacts between both sides of the Sahara with genetic influence from south of the desert still found in current-day North Africans (Idaghdour et al. 2010; Harich et al. 2010; Fadhlaoui-Zid et al. 2011; Henn et al. 2012; Arauna et al. 2017; Serra-Vidal et al. 2019; Lucas-Sánchez et al. 2021b). This is reinforced by our results from MOSAIC, which does not require a prior specification of the ancestry sources (unlike other local-ancestry-based methods), yet it infers admixture in Tunisians and Moroccans involving a non-sub-Saharan-like source and a sub-Saharan-like source. The amount of sub-Saharan-like ancestry varies significantly between individuals within the same population, as previously seen (Henn et al. 2016; Arauna et al. 2017). Most individuals exhibit a low or medium sub-Saharan-like genetic component, but a relevant percentage of samples present this component at high proportions. The observed diversity in proportions of sub-Saharan-like ancestry points to a recent gene-flow scenario, as this ancestral component might still be restricted to some parts of the population and have not had enough time to spread.

As one of the main novelties of this work, we have explored the possible origin of the sub-Saharan-like component detected in the eastern and western sides of the North African Maghreb, using the present-day populations from different regions south of the Sahara as proxies for the ancestral admixing groups. Our results reveal a differentiation between the possible origin of such gene flow in Morocco and Tunisia, especially when considering those individuals with larger sub-Saharan-like ancestry. Our analyses show evidence for two regions of origin of the admixing source coming from south of the Sahara: (1) a Western-like source present in all individuals (similar to current-day Senegambian populations), and (2) an Eastern-like source present only in Tunisians with > 50% of sub-Saharan-like ancestry (similar to current-day populations from the south-Sudan-Kenya region). Finding at least two different origins for the sub-Saharan-like component in the studied North African populations could be revealing the existence of two migrating trans-Saharan routes. The fact that the difference in admixing sources is especially relevant in samples with large sub-Saharan-like ancestry can be because older trans-Saharan migrations to Tunisia and Morocco had a similar origin, or because North African individuals have been mixing since then and thus homogenizing their sub-Saharan-like component. In contrast, a more recent migration, accounting for the large sub-Saharan-like proportions in some individuals, would have had different origins.

Dating these admixture events contributes to a better understanding of these possible two migration waves. The admixture event involving a Western-sub-Saharan-like source (in Moroccans and those Tunisians with lower sub-Saharan-like component) is dated to around the thirteenth–fourteenth centuries CE, which is consistent with the dates inferred by Arauna et al. (2017), who used a different dataset and a different approach. Historically, this period coincides with the last years of the Ghana Empire and the rise of the Mali Empire in Western Africa, matching also geographically with the inferred Senegambian-like or Western-like admixing source, especially with the Mali Empire that reached as far as the Atlantic coast (Newman 1995; Segal 2002; Falola and Heaton 2008; Messier and Miller 2015). The Ghana Empire was in fact founded by Soninke peoples, who are in the same ethno-linguistic group as the Mandenka, the Mande ethno-linguistic group. Current-day Mandenka people, also known as Malinke (Newman 1995; Eberhard et al. 2021), are actually descendants of the Mali Empire (Newman 1995; Logon 2007). These two Western African powers had strong commercial relations with North African states, including the trading of slaves from south to north of the Sahara (Lovejoy 1983; Newman 1995; Segal 2002; Harich et al. 2010), which could be the origin of the sub-Saharan-like component explored in this work as suggested by previous studies (Harich et al. 2010; Henn et al. 2012; Arauna et al. 2017). In contrast, Tunisians with > 50% of sub-Saharan-like component present a more recent inferred admixture date around the seventeenth century CE. This source has higher copying probabilities with the current-day Dinka, which is a different population from those closer to these individuals in the ASPCA (Luo, Luhya, and Bantu from Kenya), although geographically close, and they all could be related to an Eastern migration route to the north of the continent. The Dinka and the Luo are in fact closely related, both members of the Nilotic peoples group which originated in Sudan (Clark and Brandt 1984; Newman 1995). The inference of a non-sub-Saharan-like admixture source and the presence in these Tunisian individuals of the Western-like thirteenth century CE admixture event signal, although weaker than the Eastern-like, confirms that these are not sub-Saharan African individuals but admixed North African people with a strong sub-Saharan-like ancestry. Again, these results agree with those from Arauna et al. (2017), who also found evidence of admixture with a major sub-Saharan-like source dated to the seventeenth century CE, which they relate to the trans-Saharan slave trade performed by the Ottoman rulers of North Africa and the arrival of Iberian Kingdoms to the Western coast in the sixteenth century CE. The Dinka individuals in our dataset were sampled in South Sudan, where some of the major eastern historical trans-Saharan slave routes run (Lovejoy 1983; Newman 1995; Segal 2002; Harich et al. 2010), again pointing to these unwilling migrations as the origin of the sub-Saharan-like component in North Africa.

Evidence for genomic segments with origin south of the Sahara has been found in ancient North African data (van Loosdrecht et al. 2018; Serra-Vidal et al. 2019), thus previous to the start of historical trans-Saharan slave trade. However, previous work has suggested that the presence of sub-Saharan-like genomic regions prior to the beginning of the historical trans-Saharan slave trade in the current-day North Africans might be negligible in comparison to recent admixture events (Harich et al. 2010; Henn et al. 2012; Arauna et al. 2017; Lucas-Sánchez et al. 2021a). This lack of remarkable ancient gene flow from south of the Sahara in the present-day North Africans is confirmed and expanded in the present study, since our dating results point to recent admixture and our simulation analyses reject a major contribution from older trans-Saharan migrations.

The temporal trajectories of the sub-Saharan-like component are also aligned with the rest of our results and suggest a recent increase of the sub-Saharan-like genetic component in North Africa. The historically low effective population sizes observed for the sub-Saharan-like component explain the generally medium–low proportions in current-day individuals, and the increase in the more present generations is concordant with some individuals having large (and thus recent) sub-Saharan-like genetic component. In the ancestry-specific effective population size analysis, the observed growth of the sub-Saharan-like component occurs later than the inferred admixture dates in MOSAIC, around 17–18 generations ago for Tunisians and around 16–17 generations ago in Moroccans when including those samples with > 50% of sub-Saharan-like ancestry. Both can be assigned to around the fifteenth-sixteenth centuries CE. This can be the result of admixture happening over the course of multiple generations (Browning et al. 2018) and contributes to the idea of a continuous gene-flow as previously suggested (Arauna et al. 2017). Despite the fact that our confidence intervals are narrow, a larger dataset might increase the precision of this analysis.

We provide new and more detailed evidence about the origin and characteristics of the trans-Saharan migrations to North Africa using as a model two groups in the opposite extremes of Maghreb, but more North African populations should be studied with a similar approach to have a deeper understanding of the subject and explore the difference between populations, which is expected given the high cultural and demographic heterogeneity in North Africa.

## Materials and methods

### Sample collection, sequencing, and quality controls

Samples from two North African populations were newly genotyped for the present study: Tunisian individuals from the city of Tunis (*n* = 64), and Moroccan individuals from different urban areas in the country (*n* = 45): Marrakesh (*n* = 4), Casablanca (*n* = 4), Fez (*n* = 13), Nador (*n* = 3), Oujda (*n* = 2), Ouarzazate (*n* = 13), and Rabat (*n* = 6). Samples from healthy volunteers were collected for self-reported non-related individuals in these populations with appropriate informed consent. This study has been approved by our local IRB (Comitè d’Ètica de la Investigació, Parc de Salut Mar, reference 2019/8900/I) and individuals were genotyped with the Affymetrix Axiom Genome-Wide Human Origins 1 Array. Genotype calling of the raw samples was performed with the Axiom Analysis Suite 4.0 software using default settings for thresholds. Only autosomal chromosomes were included. Then, PLINK/1.9b (Purcell et al. 2007) was used to apply quality control filters and remove (1) sites with > 5% of SNP missingness, (2) individuals with > 10% of missingness, (3) SNPs failing the Hardy–Weinberg exact test with a *p* value of 10^–5^, (4) sites with a minor allele frequency (MAF) < 0.05, and (5) sites with duplicate IDs, for a final count of 425,777 sites in the dataset. Finally, relatedness between individual pairs was assessed using VCFtools 0.1.14 (Danecek et al. 2011), and 13 samples were removed so no third-degree or closer relationships remained in the dataset (all individuals have kinship coefficients < 0.0442).

These North African samples were merged with a panel of world-wide populations from different publications genotyped with the Affymetrix Axiom Genome-Wide Human Origins 1 Array (https://reich.hms.harvard.edu/datasets). Populations, number of samples, and source publications can be found in Supplementary Table 2. After merging and applying the same quality filters as previously mentioned, the final dataset contained 392,771 genome-wide SNPs and 1086 individuals from 67 populations. North African reference populations were only used in the population structure analyses, and some sub-Saharan Africa, Middle Eastern, and European populations were also excluded in some analyses as stated in the corresponding section and in Supplementary Table 2.

### Population structure analyses

Principal component analyses (PCA) were performed using the SmartPCA tool from the EIGENSTRAT stratification correction method implemented in the EIGENSOFT software package version 6.0.1 (Patterson et al. 2006). Ancestry patterns were explored applying ADMIXTURE 1.3 (Alexander et al. 2009) in unsupervised mode, exploring a range from *K* = 2 to *K* = 10 ancestral clusters with 50 independent runs for each *K* using a different random seed in each run. The upper limit of *K* = 10 was selected based on previous evidence for the studied populations (Henn et al. 2012; Arauna et al. 2017; Serra-Vidal et al. 2019; Lucas-Sánchez et al. 2021b). Cross-validation errors for each *K* were assessed at each run and mean values were calculated to determine the range with minimum error (Supplementary Fig. 16). Pong in greedy mode was used to identify common modes among the different runs for each *K* and to visualize and plot the results (Behr et al. 2016). For both PCA and ADMIXTURE, data were first pruned for linkage disequilibrium with PLINK 2.0 (Purcell et al. 2007) using sliding windows of 50 kb with a step size of 5 SNPs, and a square correlation coefficient (*r*^2^) threshold of 0.5, which kept 201,788 variant sites. PLINK 2.0 and VCFtools 0.1.14 (Danecek et al. 2011) were used to obtain intermediate files for the described analyses. Additionally, these two first exploratory analyses were used to discard some sub-Saharan African populations from further analyses, the Shaigi, the Somali, and the Afar, as they are genetically close to North Africans and their inclusion as reference sub-Saharan populations would add noise to our results.

### Local ancestry inference

To infer local ancestry in North African samples, we followed the approach presented in Martin et al. (2017). We used two different phasing tools, SHAPEIT4 (Delaneau et al. 2019) and HAPI-UR (Williams et al. 2012), independently from each other, and used the 1,000 genomes phase 3 genetic map as a reference for genetic distances (1000 Genomes Project Consortium T GP et al. 2012. Local ancestry segments were inferred with RFMix v2 (Maples et al. 2013) with recommended parameters, and setting two different ancestry sources: sub-Saharan-like ancestry, using a panel of 23 widely distributed sub-Saharan Africa populations, and non-sub-Saharan-like ancestry, using a panel of 11 Middle Eastern populations to account for the non-sub-Saharan-like genetic component (Supplementary Table 2, column “Local Ancestry Inference reference group”). We do not interpret this latest component as pure Middle Eastern ancestry, and rather as the mix of genetic components different from those south of the Sahara, which possibly include European and autochthonous North African ancestries apart from the Middle Eastern one. For this same reason, we do not call it non-African-like component. For ease of reading, we refer to this component as the “non-sub-Saharan-like component”. Some more complex analyses were repeated using (1) European, and (2) a mix of European and Middle Eastern individuals to account for the non-sub-Saharan-like ancestry, and similar results were found (see “Admixture dating and characterization”). RFMix v2 results using SHAPEIT4 and HAPI-UR phased data, respectively, exhibit extremely similar results. Per-individual average ancestry proportions were calculated by averaging across all chromosomes.

### Ancestry-specific principal component analysis

To plot principal components corresponding to local ancestry segments of specific ancestries, we followed the local-ancestry-based method developed by Browning et al. (2016), which in turn was based on another method presented by Moreno-Estrada et al. (2013), called ancestry-specific principal component analysis (ASPCA). ASPCA provides information about the genetic similarities between local-ancestry-inferred segments of a specific ancestry and actual individuals from the region of origin of such ancestry. This can be extrapolated as closeness between the ancestors of the admixed population and those of current-day reference individuals, which can be because the original admixing population came from the same geographical region as the closest individuals in the ASPCA, or because the admixed group and its closest reference samples in the ASPCA share a common ancestor elsewhere in the geography. We applied ancestry-specific masking to the local-ancestry assessed phased samples (see “Ancestry-specific effective population size” below) setting to missing all alleles not corresponding to haplotypes assigned to the sub-Saharan-like ancestry. The same process was performed to leave as non-missing those alleles corresponding to haplotypes assigned to the non-sub-Saharan-like ancestry. Data were pruned for linkage disequilibrium using the same parameters as in the PCA. We then applied principal components on a per-haplotype basis to the masked haplotypes using multidimensional scaling (MDS) with Euclidean distance, treating alleles as 0 s and 1 s. When selecting the North African samples to be plotted, we applied a threshold to the amount of the selected ancestry component. We applied six different increasing ancestry thresholds: 0%, 10%, 20%, 30%, 40%, and 50%, and produced the corresponding principal components plots. We conducted two sets of analysis within the ASPCA method, as described below.

As a quality check, we first plotted all ancestry-masked samples (for both ancestries) with the whole set of selected reference sub-Saharan African and non-sub-Saharan African populations. In this case, each North African individual produced four dots on the plot, two haplotypes per each ancestry. Samples retaining only the inferred sub-Saharan-like ancestry cluster with the reference sub-Saharan African samples, and those retaining the non-sub-Saharan-like component cluster with Middle Eastern reference samples. This is, haplotypes masked to keep only sites assigned to a specific ancestry plotted together with individuals from the corresponding ancestry, thus confirming the robustness of the ancestry-specific masking performed (Supplementary Fig. 16).

Then, we kept only reference individuals from south of the Sahara and plotted them jointly with North African haplotypes keeping only those sites assigned to the sub-Saharan-like ancestry to look for the closest sub-Saharan African populations to the sub-Saharan-like ancestry segments in North African individuals following the commands from Browning et al. (2016).

As a statistical proof of the closeness between North African haplotypes and sub-Saharan African populations in the ancestry-specific PCA, we calculated the average Euclidean distance between each North African haplotype and each sub-Saharan African population, and then computed *t* tests as stated in Supplementary Note 1. The haplotype-population distance was calculated averaging across all the distances in the PCA between each given North African haplotype and each haplotype of one African population south of the Sahara. This was repeated for all possible pairs of North African haplotypes and sub-Saharan African populations in the PCA with a threshold of 50% of sub-Saharan-like ancestry.

### Admixture dating and characterization

Dating and characterization of the admixture events were performed using MOSAIC (Salter-Townshend and Myers 2019). MOSAIC applies a double-layer Hidden Markov model to obtain information about local-ancestry and copying probabilities and learn about the characteristics of the admixture event leading to the studied admixed populations (Salter-Townshend and Myers 2019). Among these characteristics are the possible composition of the ancestral admixing groups using current-day populations as reference. We again phased the data using Beagle 4.1 (Browning and Browning 2007), but we kept only our newly genotyped North African samples (Tunisians and Moroccans), and reference samples from selected sub-Saharan Africa, Middle Eastern, and European populations (see Supplementary Table 2, column “MOSAIC role”). MOSAIC was then run following the recommendations of the software’s authors. We used 50 donors per grid point, a maximum of 20 donors per panel, and 0.9 as the threshold of copying probability before stopping. We run MOSAIC a total of six times, three per population, one with all individuals, one with those individuals with > 50% of sub-Saharan-like ancestry, and one with individuals with < 50% of sub-Saharan-like ancestry.

For the dates of admixture, we computed the confidence intervals with the “bootstrap_individuals_coanc_curves” function in MOSAIC running 1000 bootstrap runs and plotting the bootstrapped date values as a density distribution.

The same approach was performed using (1) only references from sub-Saharan Africa and the Middle East, and (2) only references from sub-Saharan Africa and Europe, with similar results (data not shown).

Concordance between RFMix v2 and MOSAIC was assessed by comparing the proportions of sub-Saharan-like ancestry inferred by both methods in each individual and fitting a linear model. For MOSAIC, the sub-Saharan-like component was assigned to that component of the two inferred with more sub-Saharan populations among the top groups in the copying probabilities list. The correlation coefficient (*r*^2^) and *f*-statistic *p* value were recorded. As an additional check, a *t* test was performed comparing the two sets of inferred sub-Saharan-like proportions.

### Admixture simulation and segment-length distribution

Since haplotype-based methods are biased to detect more recent admixture events and older admixture events might be underestimated, we simulated different admixture scenarios and then compared their resulting distribution of sub-Saharan-like segments length to that of our observed data. Simulations were performed with the forward-time simulation approach AdmixSim (Yang et al. 2020), parallelizing the analyses by chromosome and setting the chromosome length at 500 to cover all of our data. We first simulated a one-wave event with a model of admixture in 3:1 proportion between 126 French and 126 Yoruba haplotypes, respectively, with a permanent population size of 100, and sampling 40 individuals from the resulting admixed population. We performed this approach three times, setting the generations since admixture at 25, 50 and 100. Then, in the simulated populations admixed 50 and 100 generations ago, we simulated a second admixture event with Yoruba 25 generations ago using the same proportions and parameters as described previously but using 80 haplotypes for each source population.

We generated VCF files for the five simulated populations from the haplotype files outputted by AdmixSim to perform local ancestry inference as described above and compared the length distribution of those segments assigned to a sub-Saharan-like ancestry to the same distribution in our observed data. Statistical analysis of these comparisons was performed with the Kolmogorov–Smirnov test using the “ks.boot” function of the R package “Matching”, performing 1000 bootstrap repetitions. Tests were performed in the comparison of the complete distributions and in the distribution of segments ≤ 10 cM, as this is the range where most segments were found.

### Ancestry-specific effective population size

To assess the composition of the admixing populations prior to the admixture, we combined LAI and identity-by-descent (IBD) information, as in the method developed by Browning et al. (2018). Following this method, we detected IBD segments in phased data from Tunisian and Moroccan individuals, inferred local ancestry using RFMix, and combined both results to assign a continental ancestry to each IBD segment. Then, we performed effective population size determination in these ancestry-labeled IBD segments using IBDNe. Data were first phased with Beagle 4.1 (Browning and Browning 2007), and then, IBD segments were detected in the phased data using the haplotype-based IBD detection method Refined-IBD (Browning and Browning 2013) using default settings and applying an IBD segment-length threshold of 2 cM. Merge-IBD (Browning et al. 2018) was used to fill possible gaps between IBD segments that result from genotype and haplotype phase errors, filling gaps if those were less than 0.6 cM long and there was at most one pair of genotypes inconsistent with IBD in the gap.

Local ancestry was inferred using RFMix v1.5.4 (Maples et al. 2013) in the phased data with the parameters recommended in the documentation, and setting two groups of populations as references for two different ancestries, a sub-Saharan-like ancestry and a non-sub-Saharan-like ancestry. Specific populations in each group can be found in Supplementary Table 2 (see column “Local Ancestry Inference reference group”). Rephasing was applied to the local ancestry haplotypes to match the IBD segment haplotypes using the rephasevit tool from Browning et al. (2018). Following the same reference publication, each IBD segment was assigned ancestry proportions using the phase-adjusted local ancestry called for the corresponding haplotypes. Each IBD was assigned ancestry proportions equal to the mean called local ancestry from the two corresponding haplotypes. With ancestry-labeled IBD segments, we run the IBDNe software (Browning and Browning 2015) to calculate the effective population size from IBD segments per population (Tunisians and Moroccans separately). We used a threshold of 2 cM for IBD length, adjusted the number of haplotype pairs of each ancestry as in Browning et al. (2018), and run the program in three different settings: considering all IBDs (general effective population size), only considering the sub-Saharan-like ancestry proportions of each segment, and only considering the non-sub-Saharan-like ancestry proportions of each segment (ancestry-specific effective population size). However, as exposed in Browning et al. (2018), for the ancestry-specific effective population size, the full length of IBD segments is also needed to estimate coalescence times. This is achieved by randomly assigning each IBD segment to an ancestry with probabilities equal to that ancestry proportions in the segment, resulting in an unbiased estimate of the observed total length of IBDs corresponding to each ancestry. IBDNe performs bootstrap resampling of chromosomes with replacement to obtain confidence intervals for the estimated effective population size trajectories. We used the default 80 bootstrap replicates and show in our plots the 2.5th and 97.5th percentiles to represent the precision of effective population size estimates.

Effective population size was calculated a total of five times per North African population: (1) general ancestry-unaware effective population size, (2, 3) ancestry-specific effective population size for each of the two ancestries including all individuals in that population, and (4, 5) ancestry-specific effective population size for each of the two ancestries excluding individuals with > 50% of sub-Saharan-like ancestry.

In addition, we also calculated the effective population size of all African individuals from south of the Sahara present in our dataset as a whole to compare it to the trajectory of the sub-Saharan-like ancestry of North African populations. We used the same method described here but taking only the IBDs from African samples from south of the Sahara and without inferring local ancestry.

## Supplementary Information

Below is the link to the electronic supplementary material.

Supplementary file1 (DOCX 8922 KB)

Supplementary file2 (XLSX 24 KB)

## Data Availability

Tunisian and Moroccan genotypes are deposited at EGA Accession Number: EGAS00001006427.
