# Supplementary material for: The genomic analysis of current-day North African populations reveals the existence of trans-Saharan migrations with different origins and dates
Source: Hum Genet. 2022 Nov 28;142(2):305–20. doi: 10.1007/s00439-022-02503-3 (PMC9918576; doi:10.1007/s00439-022-02503-3)
Supplement: Supplementary file 1 — Supplementary file1 (DOCX 8922 KB) [file 439_2022_2503_MOESM1_ESM.docx]

**The genomic analysis of current-day North African populations reveals the existence of trans-Saharan migrations with different origins and dates.**

*Human Genetics*

Marcel Lucas-Sánchez^1^ (ORCID: 0000-0001-6741-3959), Karima Fadhlaoui-Zid^2,3^ (ORCID: 0000-0002-8980-9716), David Comas^1,*^ (ORCID: 0000-0002-5075-0956)

^1^ Institut de Biologia Evolutiva (CSIC-Universitat Pompeu Fabra), Departament de Medicina i Ciències de la Vida, Universitat Pompeu Fabra, Barcelona, Spain

^2^ Laboratory of Genetics, Immunology, and Human Pathologies, Faculty of Science of Tunis,

University of Tunis El Manar, Tunis, Tunisia

^3^ College of Science, Department of Biology, Taibah University, Al Madinah Al Monawarah,

Saudi Arabia

^*^ Correspondence author – e-mail: david.comas@upf.edu

**
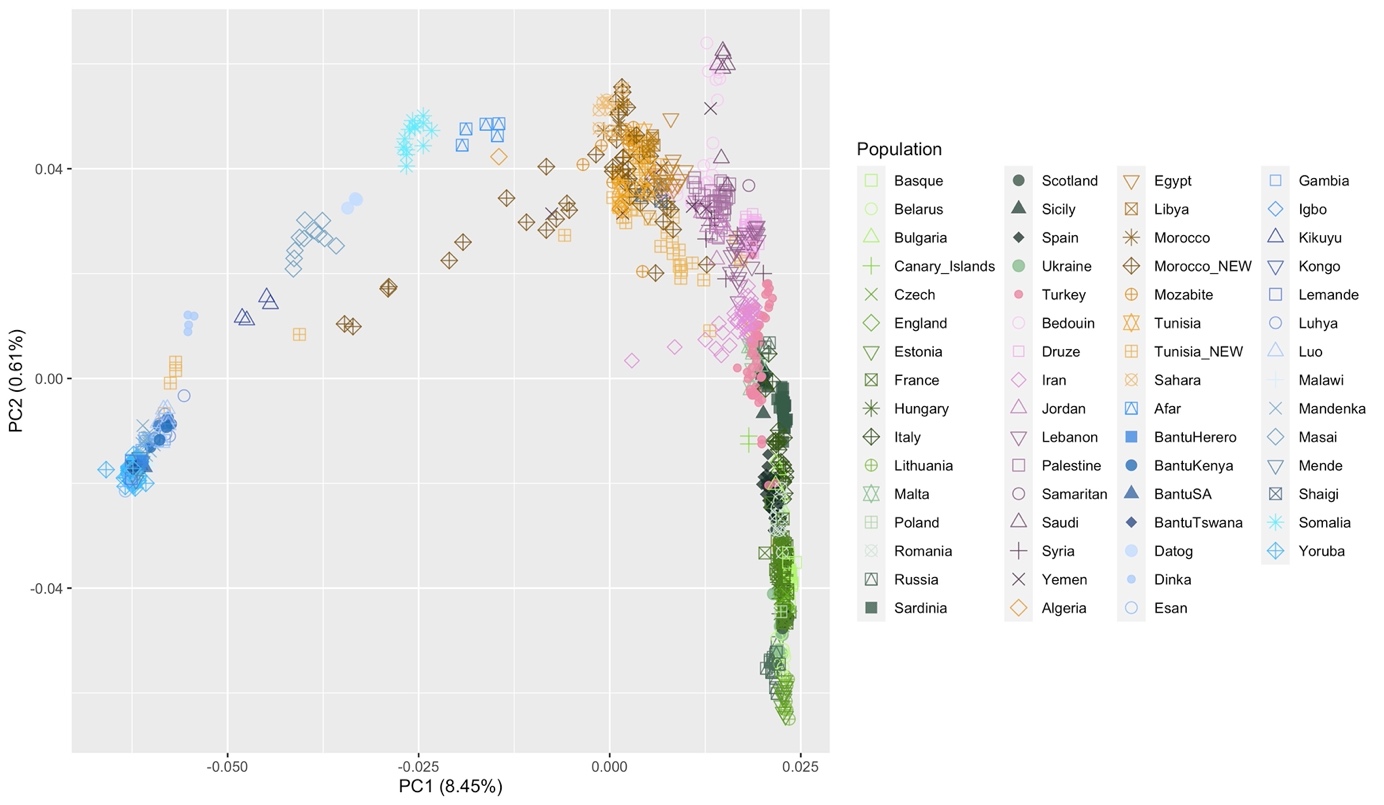
**

**Supplementary Fig. 1 Principal Component Analysis removing outliers**

Principal component analysis of the newly genotyped Tunisian and Moroccan samples (with the “NEW” label in the legend) along with a panel of reference populations from Europe (in green), the Middle East (in pink and purple), North Africa (in yellow and brown), and sub-Saharan Africa (in blue). Some individuals present in Figure 1a have been removed from the plot due to their outlier position according to the SmartPCA algorithm.


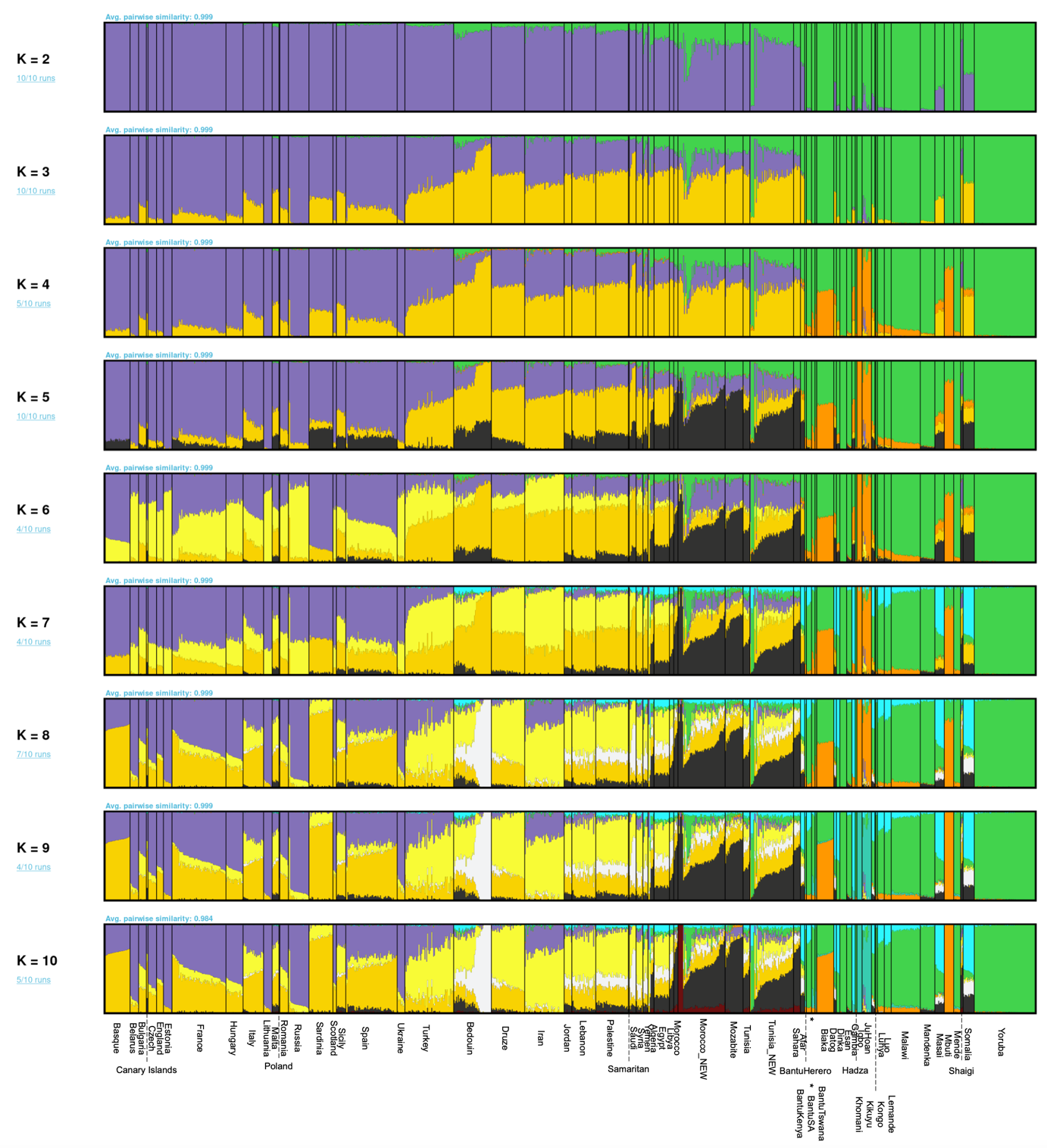


**Supplementary Fig. 2 ADMIXTURE analysis from K = 2 to K = 10**

For each K, depicted are the number of different runs giving the shown result, and the average pairwise similarity between runs. Some population names have been separated from the bottom of the plot for ease of reading. Lowest cross-validation error is K=7 (Supplementary Fig. 17).


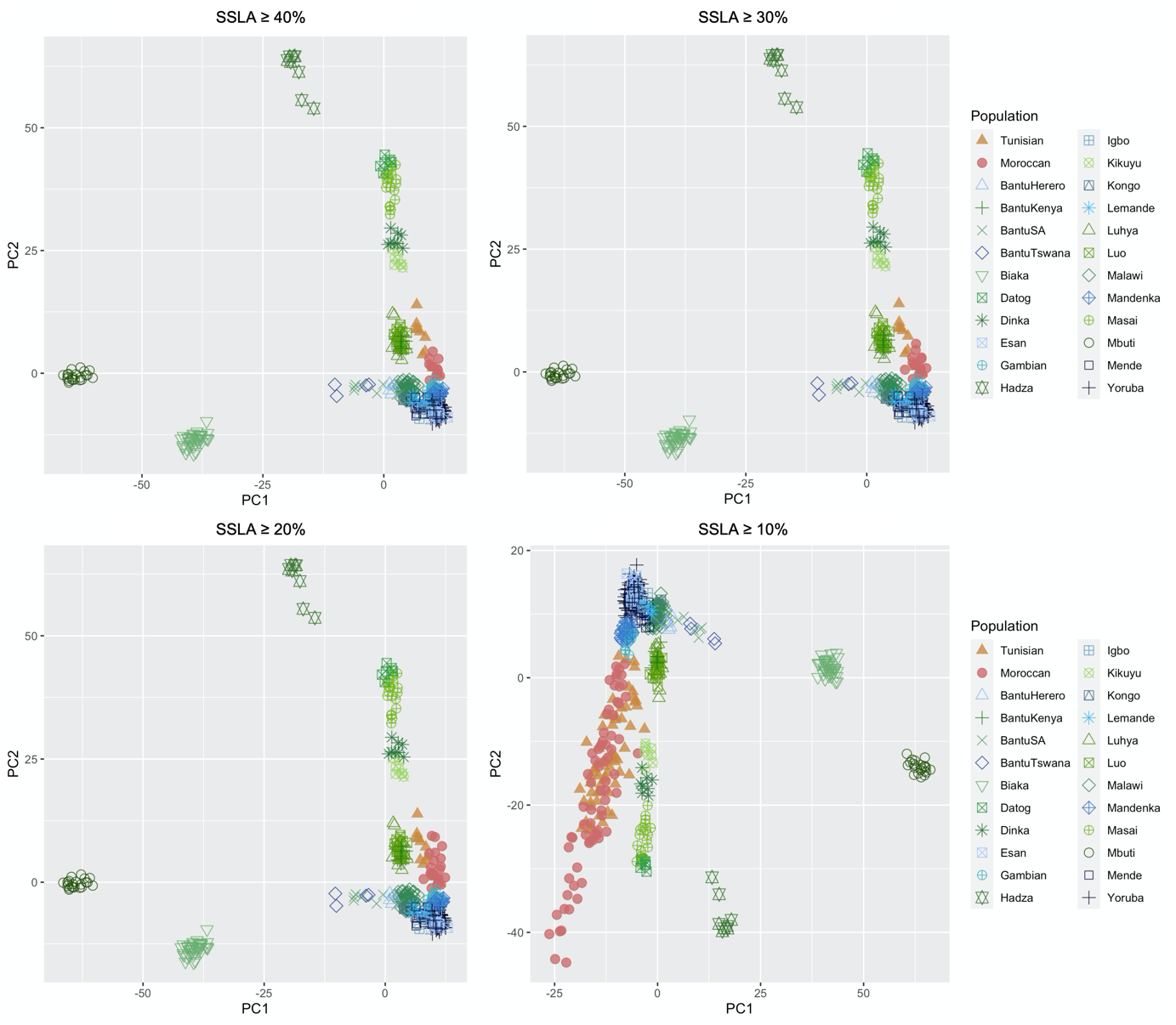


**Supplementary Fig. 3 AS-PCA with thresholds of 40%, 30%, 20% and 10% for the proportion of sub-Saharan-like ancestry.**

Ancestry-specific PCs for the sub-Saharan-like ancestry. Plotted are North African haplotypes masked to keep only sites assigned to a sub-Saharan-like ancestry and whose sub-Saharan-like ancestry is inferred to be at least 40% (top-left panel), 30% (top-right), 20% (bottom-left panel), or 10% (bottom-right), and reference sub-Saharan African populations’ haplotypes. In green are haplotypes from eastern-sub-Saharan populations and in blue are haplotypes from western-sub-Saharan populations.

**Supplementary Fig. 4 Ancestry-aware coancestry curves for Tunisians.**

Admixture dates are estimated by coancestry curve fitting as described in Salter-Townshend and Myers (2019). The y-axis shows the ratio of probabilities of pairs of local ancestries at each given genetic distance (x-axis, in centi-Morgans). On top of each panel is shown the pair of examined ancestries (1: sub-Saharan-like ancestry; 2: non-sub-Saharan-like ancestry), and, in parenthesis the estimated number of generations since admixture, which corresponds to the curve decay parameter.

**Supplementary Fig. 5 Admixture dates inferred by MOSAIC’s ancestry-aware coancestry curves and bootstrap reanalysis for the whole Tunisian population.**

Each panel shows the distribution of admixture dates in generations before present (x-axes) obtained by bootstrapping by individual the ancestry-aware coancestry curves inferred by MOSAIC. The central panel is constructed taking into account admixture between both inferred sources, while the lateral panels take only into account one of the ancestry sources (sub-Saharan-like on the left and non-sub-Saharan-like on the right).

**Supplementary Fig. 6 Ancestry-aware coancestry curves for Tunisians with >50% of sub-Saharan-like ancestry.**

Admixture dates are estimated by coancestry curve fitting as described in Salter-Townshend and Myers (2019). The y-axis shows the ratio of probabilities of pairs of local ancestries at each given genetic distance (x-axis, in centi-Morgans). On top of each panel is shown the pair of examined ancestries (1: sub-Saharan-like ancestry; 2: non-sub-Saharan-like ancestry), and, in parenthesis the estimated number of generations since admixture, which corresponds to the curve decay parameter.

**Supplementary Fig. 7 Ancestry-aware coancestry curves for Tunisians with <50% of sub-Saharan-like ancestry.**

Admixture dates are estimated by coancestry curve fitting as in Salter-Townshend and Myers (2019). The y-axis shows the ratio of probabilities of pairs of local ancestries at each given genetic distance (x-axis, in centi-Morgans). On top of each panel is shown the pair of examined ancestries (1: sub-Saharan-like ancestry; 2: non-sub-Saharan-like ancestry), and, in parenthesis the estimated number of generations since admixture, which corresponds to the curve decay parameter.

**Supplementary Fig. 8 Ancestry-aware coancestry curves for Moroccans.**

Admixture dates are estimated by coancestry curve fitting as described in Salter-Townshend and Myers (2019). The y-axis shows the ratio of probabilities of pairs of local ancestries at each given genetic distance (x-axis, in centi-Morgans). On top of each panel is shown the pair of examined ancestries (1: sub-Saharan-like ancestry; 2: non-sub-Saharan-like ancestry), and, in parenthesis the estimated number of generations since admixture, which corresponds to the curve decay parameter.

**Supplementary Fig. 9 Ancestry-aware coancestry curves for Moroccans with >50% of sub-Saharan-like ancestry.**

Admixture dates are estimated by coancestry curve fitting as described in Salter-Townshend and Myers (2019). The y-axis shows the ratio of probabilities of pairs of local ancestries at each given genetic distance (x-axis, in centi-Morgans). On top of each panel is shown the pair of examined ancestries (1: sub-Saharan-like ancestry; 2: non-sub-Saharan-like ancestry), and, in parenthesis the estimated number of generations since admixture, which corresponds to the curve decay parameter.

**Supplementary Fig. 10 Ancestry-aware coancestry curves for Moroccans with <50% of sub-Saharan-like ancestry.**

Admixture dates are estimated by coancestry curve fitting as described in Salter-Townshend and Myers (2019). The y-axis shows the ratio of probabilities of pairs of local ancestries at each given genetic distance (x-axis, in centi-Morgans). On top of each panel is shown the pair of examined ancestries (1: sub-Saharan-like ancestry; 2: non-sub-Saharan-like ancestry), and, in parenthesis the estimated number of generations since admixture, which corresponds to the curve decay parameter.

**
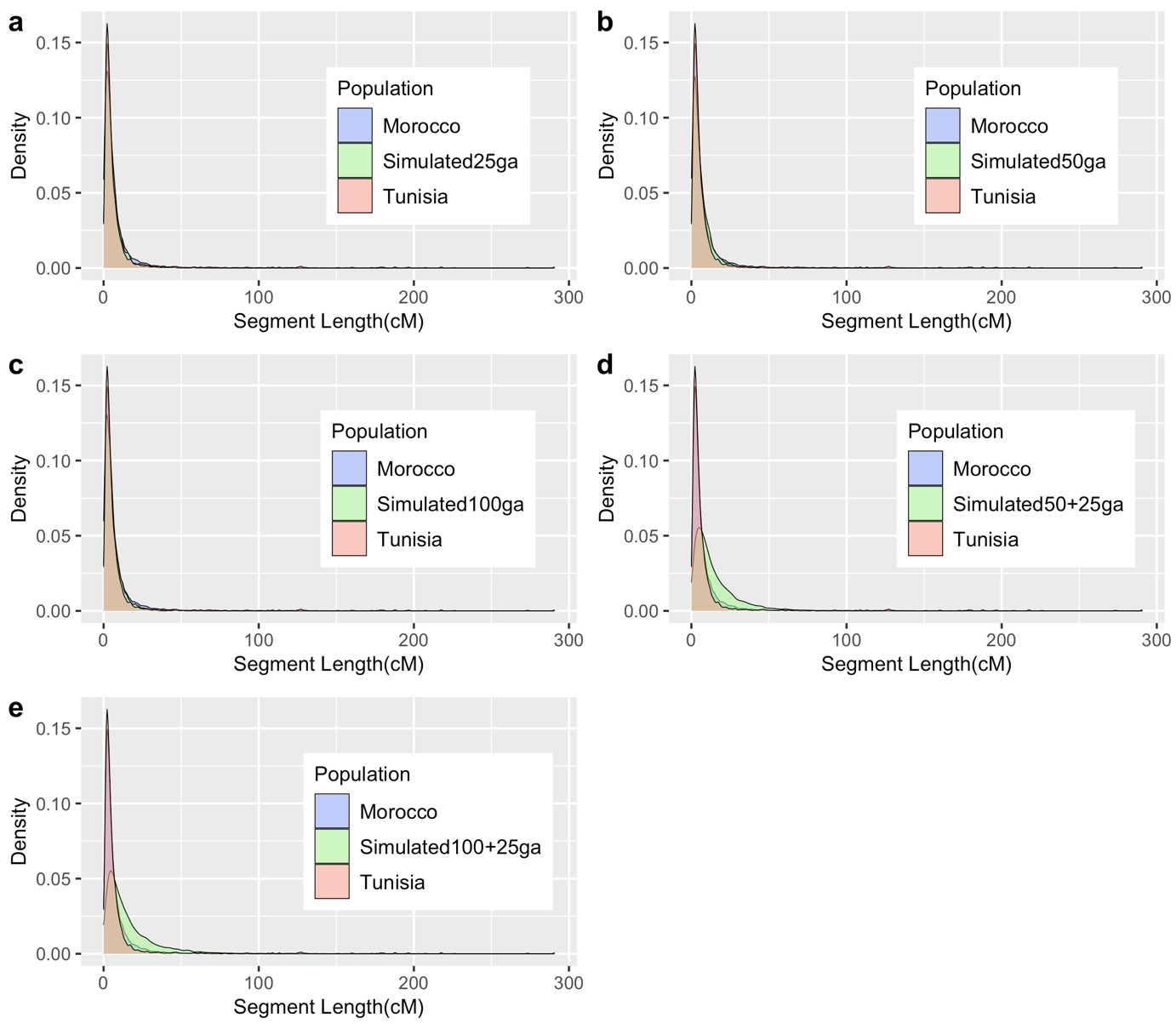
**

**Supplementary Fig. 11 Sub-Saharan-like segments length distribution compared between observed and simulated data.**

Sub-Saharan-like segments are taken from the local ancestry inference results in our two observed populations and the five simulated populations. Each panel shows the density distribution of those segment’s length for the two observed populations and one of the simulated groups with one-wave admixture (a-c) or two-wave admixture (d,e). In legends, ga stands for generations ago.

**
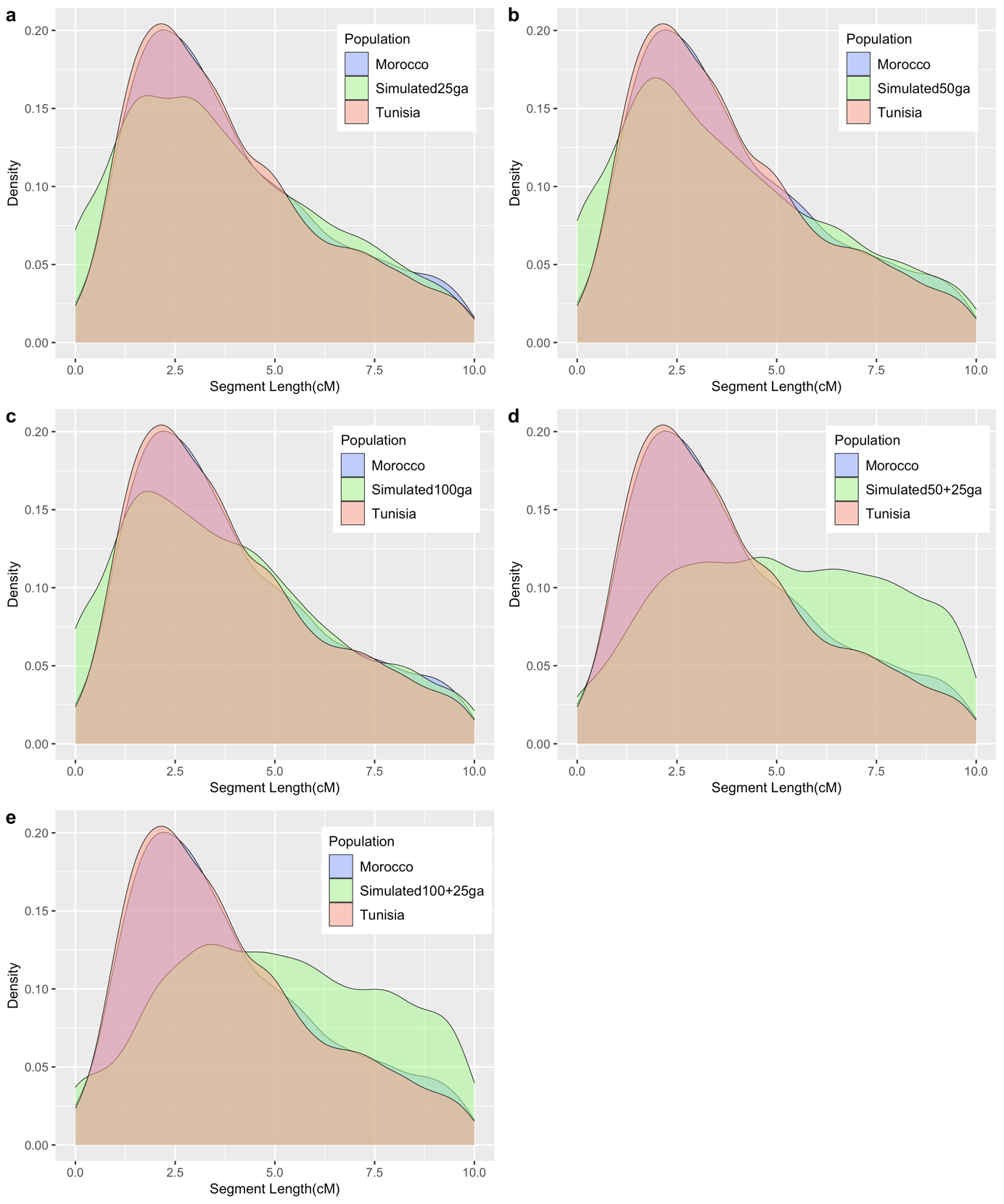
**

**Supplementary Fig. 12 Sub-Saharan-like segments length distribution for segments ≤ 10cM compared between observed and simulated data.**

Sub-Saharan-like segments are taken from the local ancestry inference results in our two observed populations and the five simulated populations. Only those segments with length ≤ 10cM are used here. Each panel shows the density distribution of those segment’s length for the two observed populations and one of the simulated groups with one-wave admixture (a-c) or two-wave admixture (d,e). In legends, ga stands for generations ago.


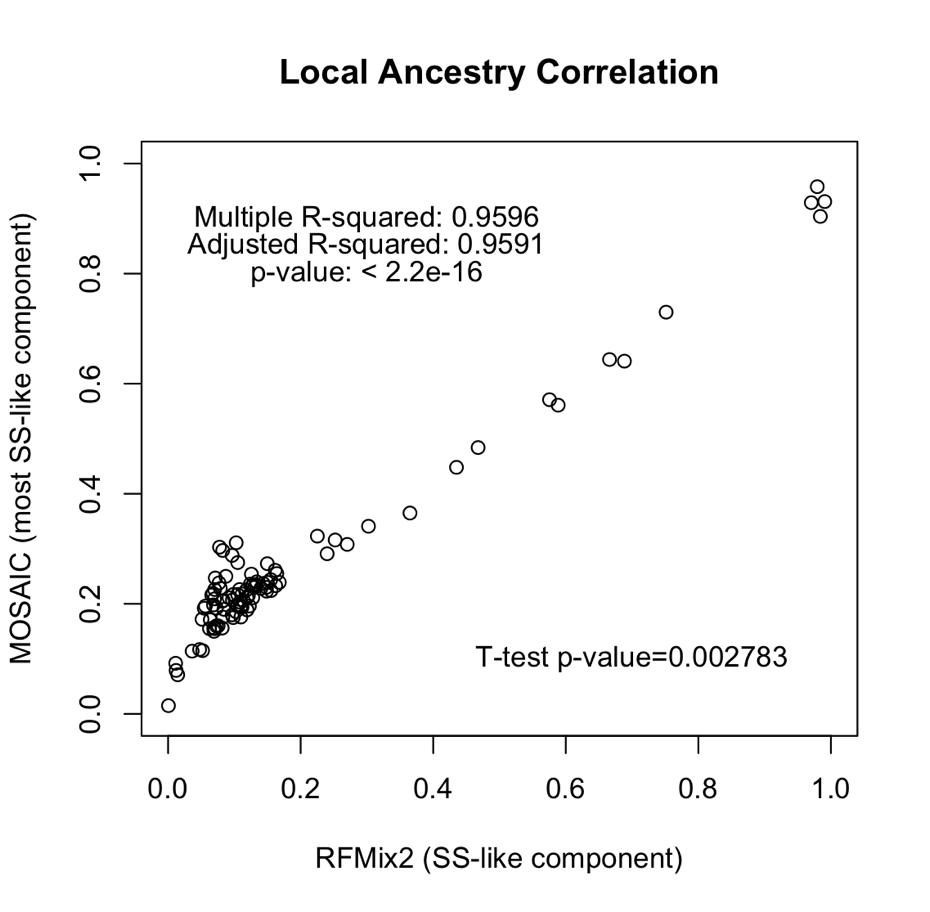


**Supplementary Fig. 13 Concordance between RFMix v2 and MOSAIC regarding inferred sub-Saharan-like ancestry**

Each dot represents an individual, with an x-coordinate equal to the proportion of sub-Saharan-like component inferred by RFMix v2, and a y-coordinate equal to the proportion of the most sub-Saharan-like component inferred by MOSAIC, i.e., the component with more sub-Saharan populations in the groups with higher copying probabilities. Inside the plot are stated, in the top left corner, the multiple and adjusted r^2^ for the fitted linear model and the p-value for the model’s F-statistic. In the bottom right corner, it is stated the p-value for the T-test comparing both groups of inferred sub-Saharan-like ancestry.

**Supplementary Fig. 14 Total effective population size**

Effective population size (N_e_) is plotted on a log scale (y-axes) and divided in overall Tunisian N_e_ (in blue), overall Moroccan N_e_ (in red), and the N_e_ joining both North African populations as one (in black). The x-axes show generations before the present.

**
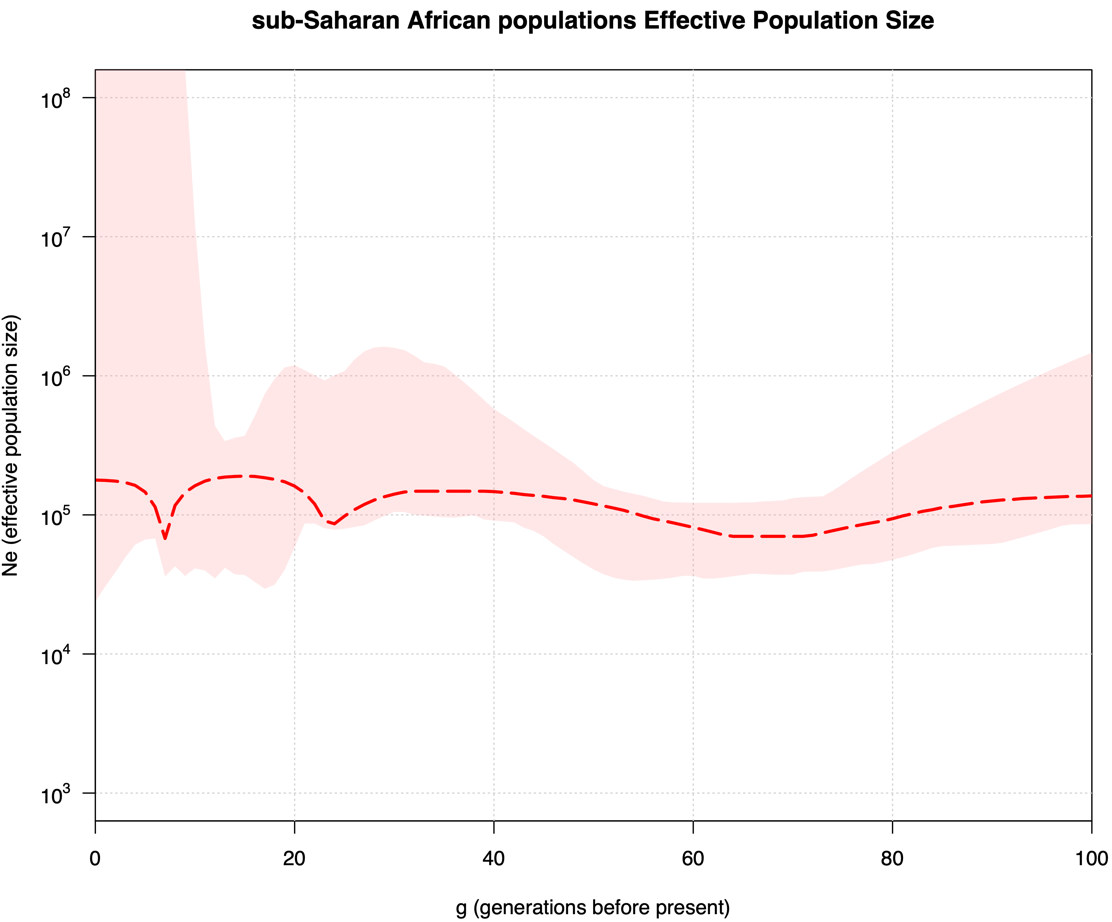
**

**Supplementary Fig. 15 Effective population size in the African individuals from south of the Sahara present in our dataset**

Effective population size (N_e_) of all African individuals from south of the Sahara present in our dataset grouped as a single population is plotted on a log scale (y-axes) with the x-axes showing generations before the present.


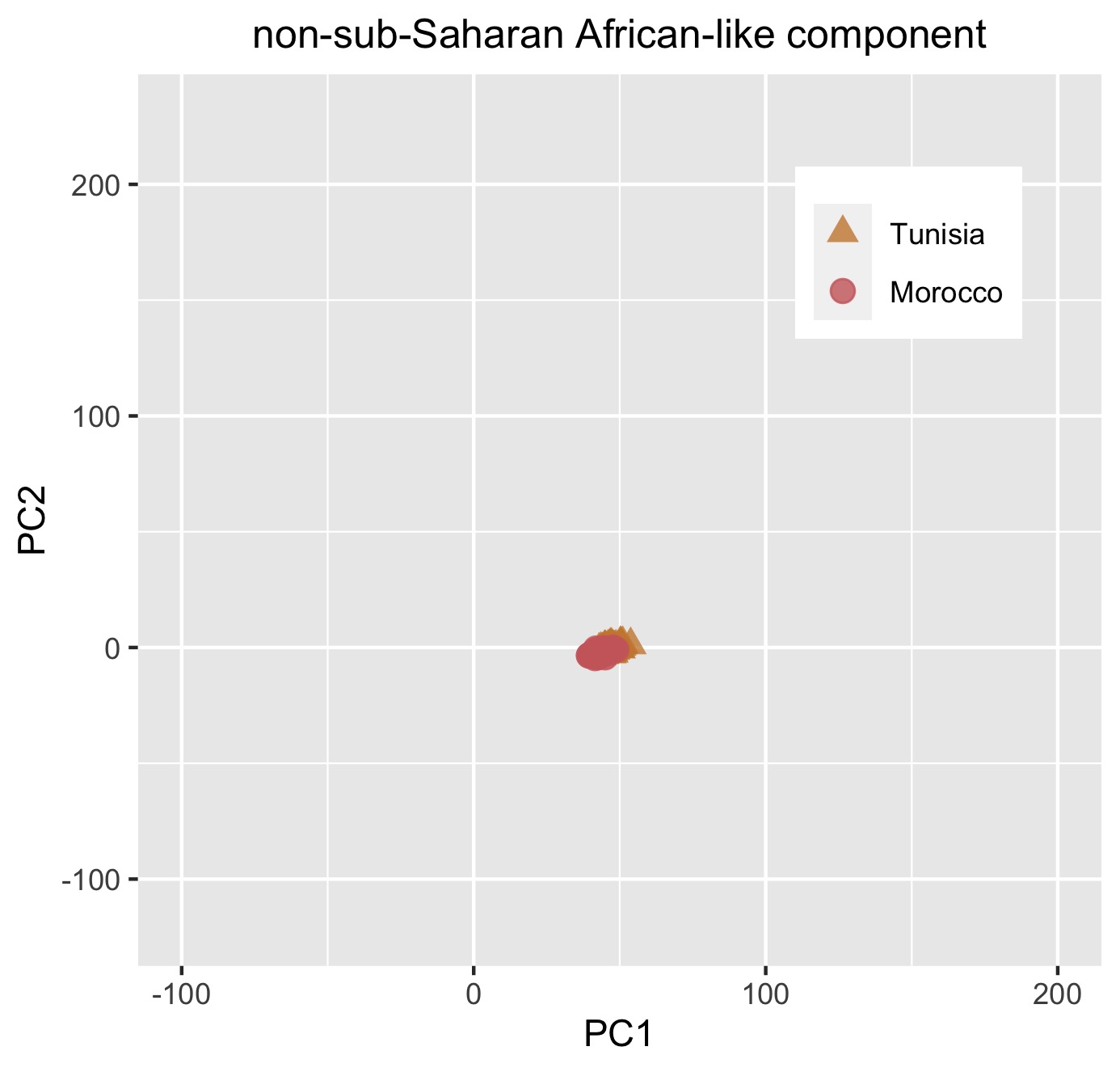

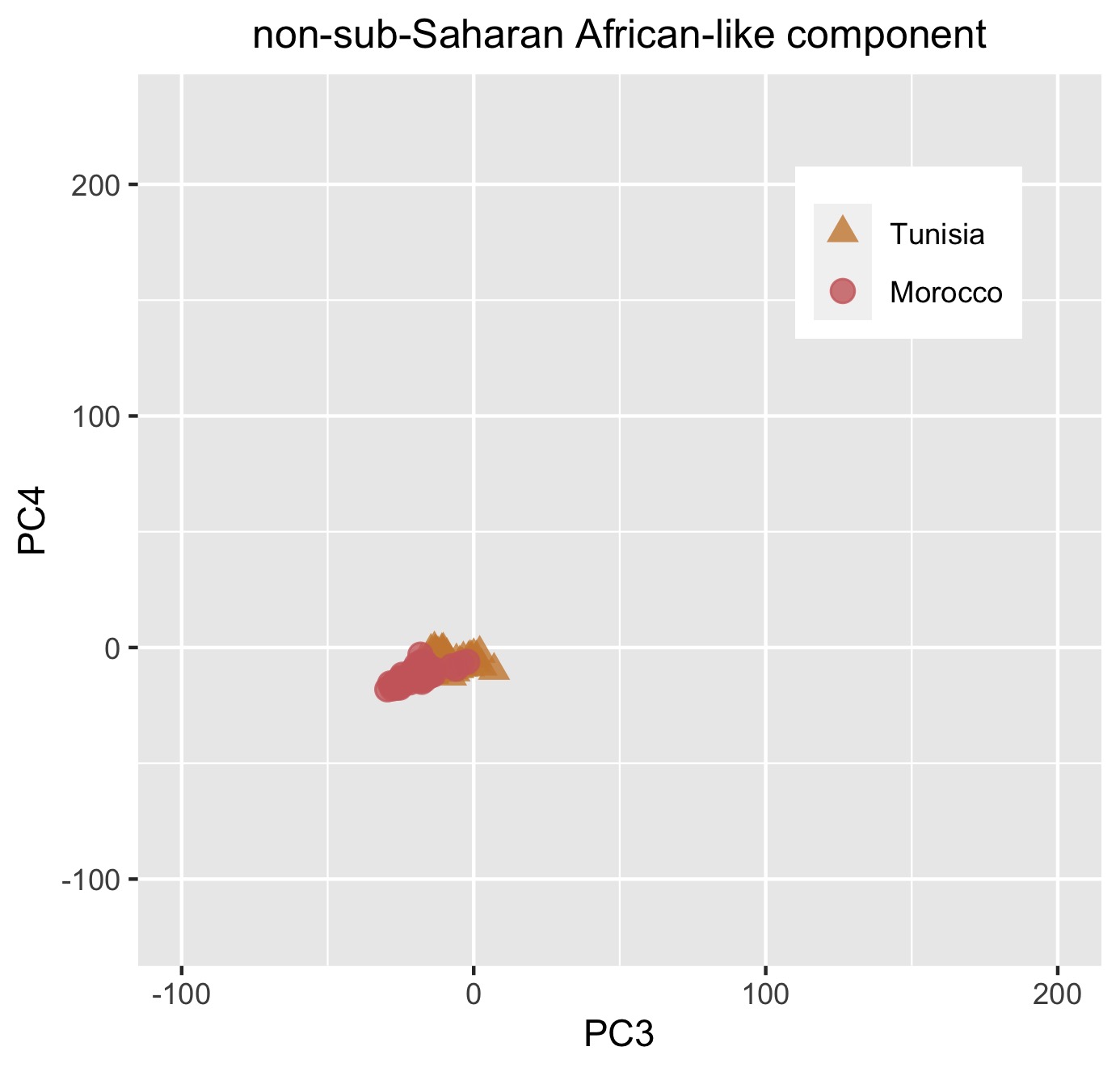

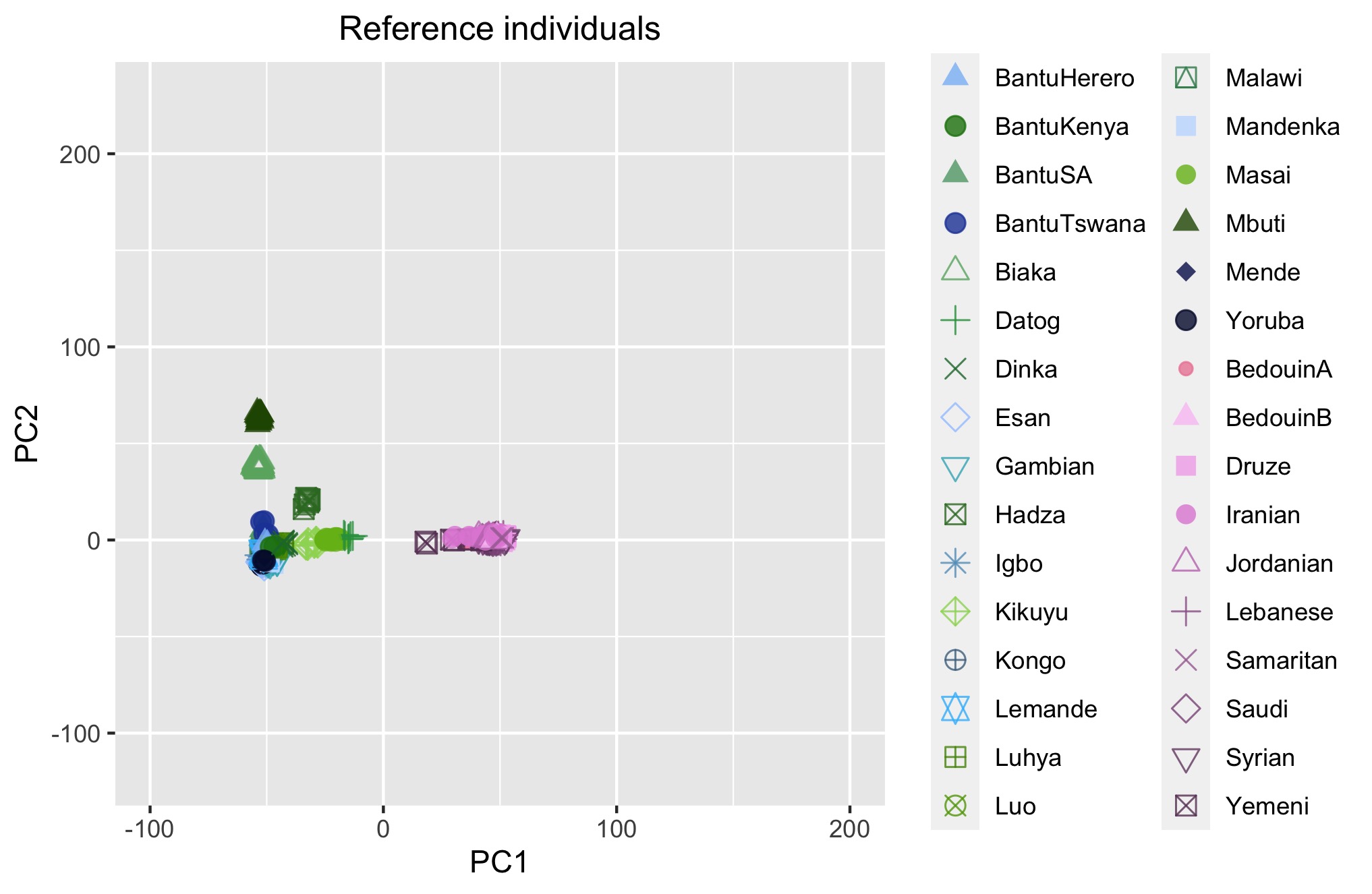

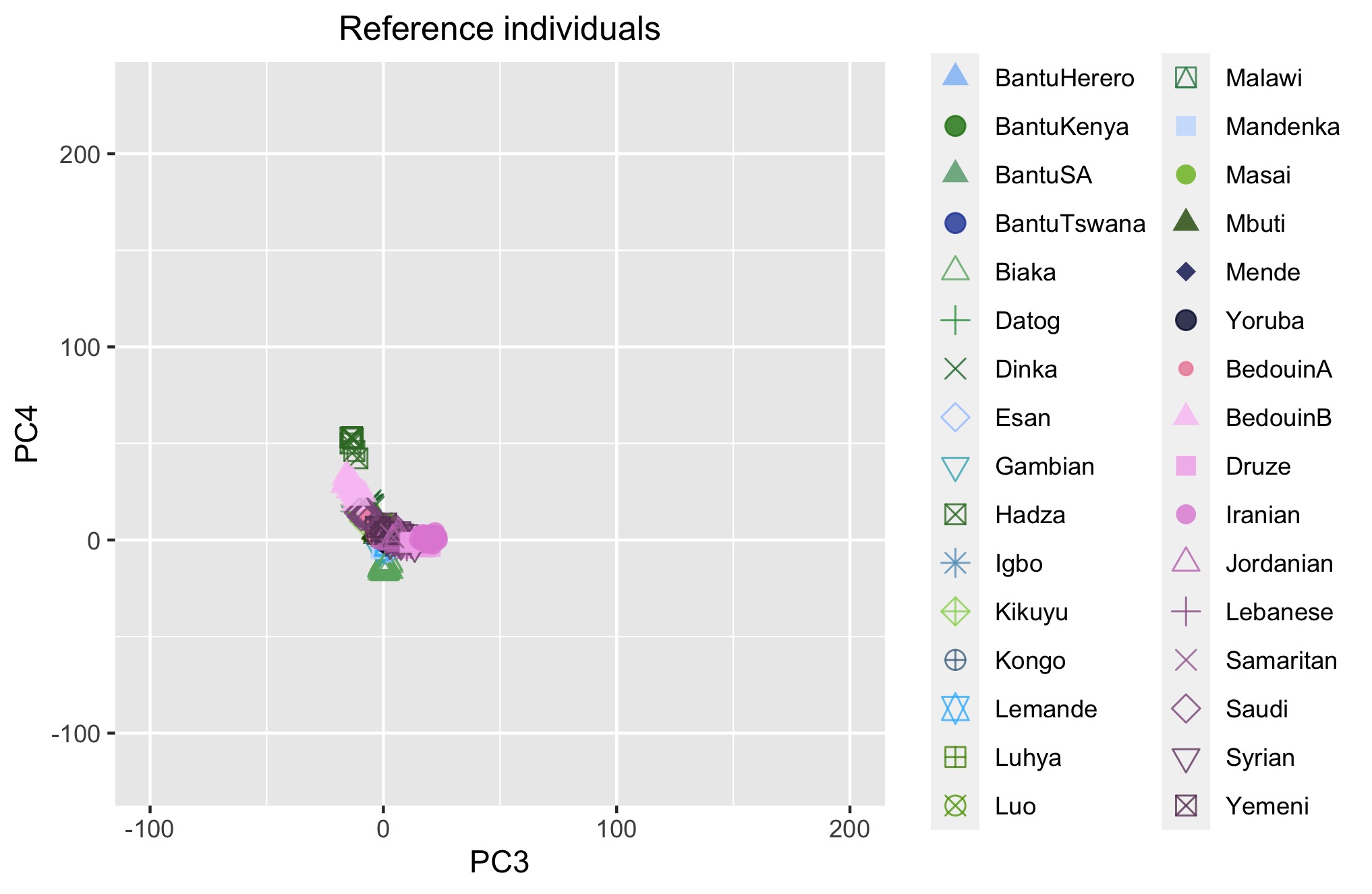

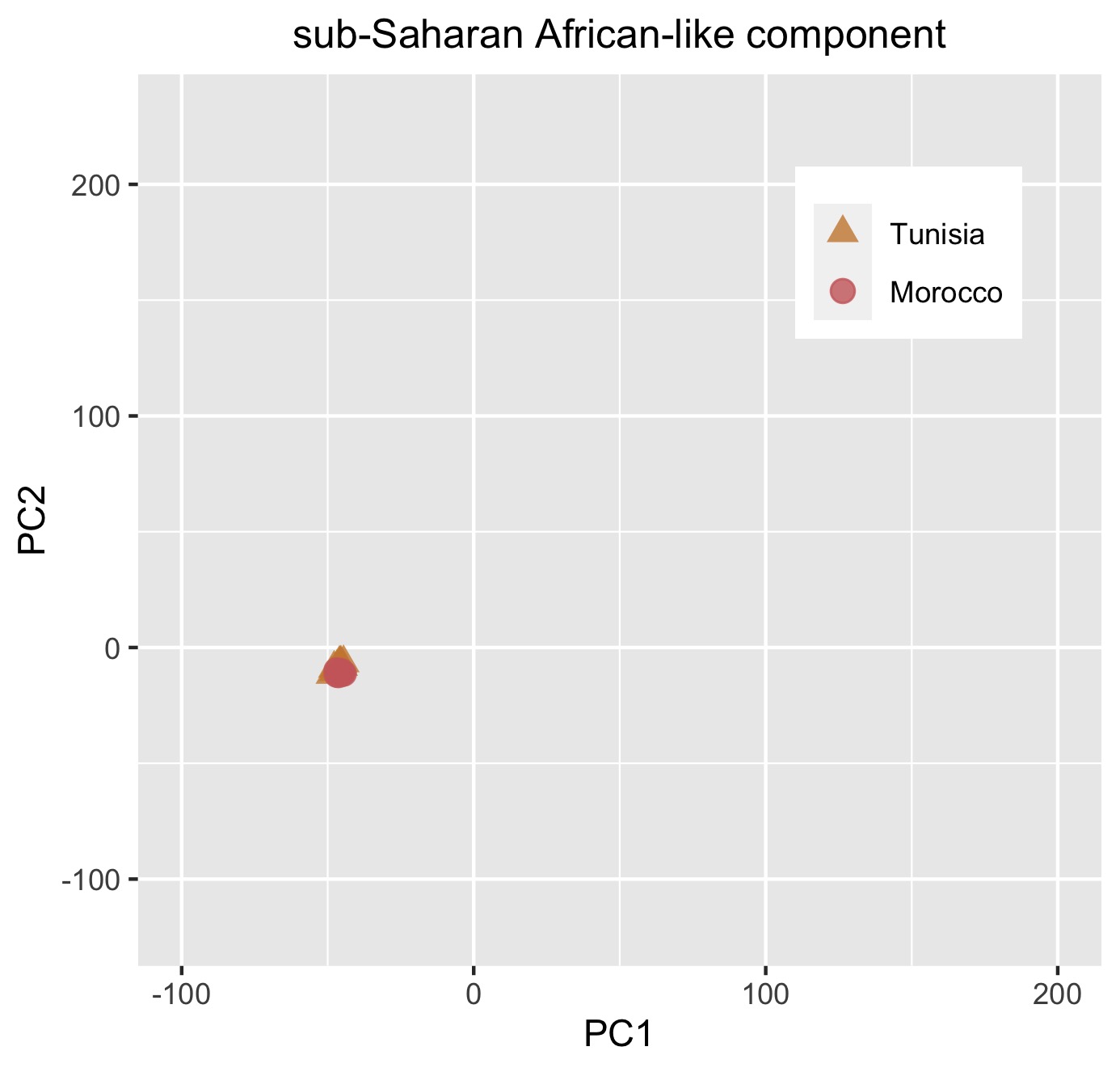

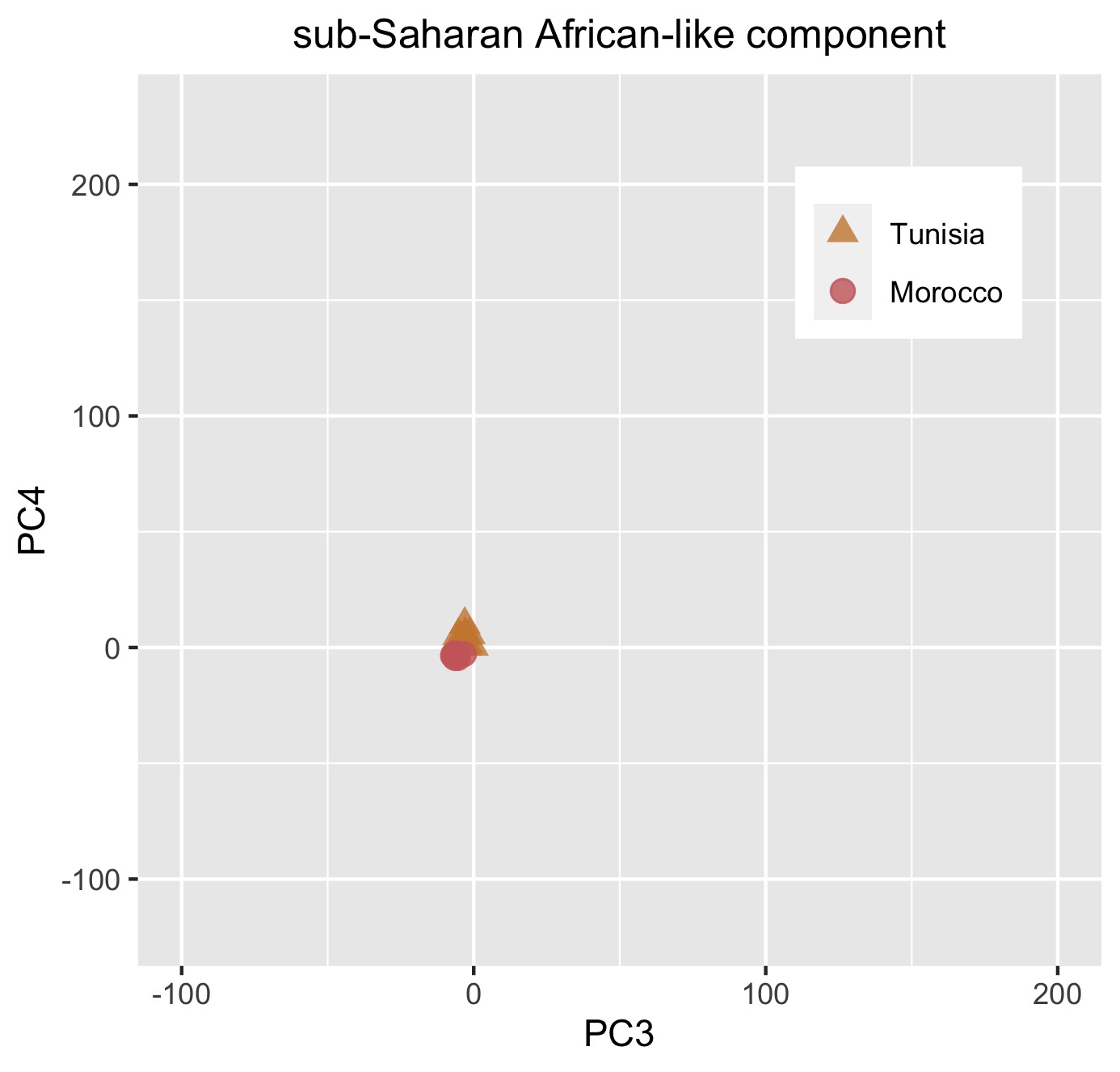


**Supplementary Fig. 16 Ancestry-specific PCA with both ancestries.**

Ancestry-specific PCA including all reference and ancestry-labelled admixed haplotypes. The threshold used for admixed individuals was set at 50%, so only individuals with at least half of their ancestry profile assigned to each specific ancestry are plotted. The first two columns show the two ancestral components inferred in the admixed individuals. The third column shows the reference individuals. PC coordinates for the three columns are calculated together and may be compared. Haplotypes are plotted, so each reference individual generates two dots, and each admixed individual generates four dots (two dots per ancestry).

**
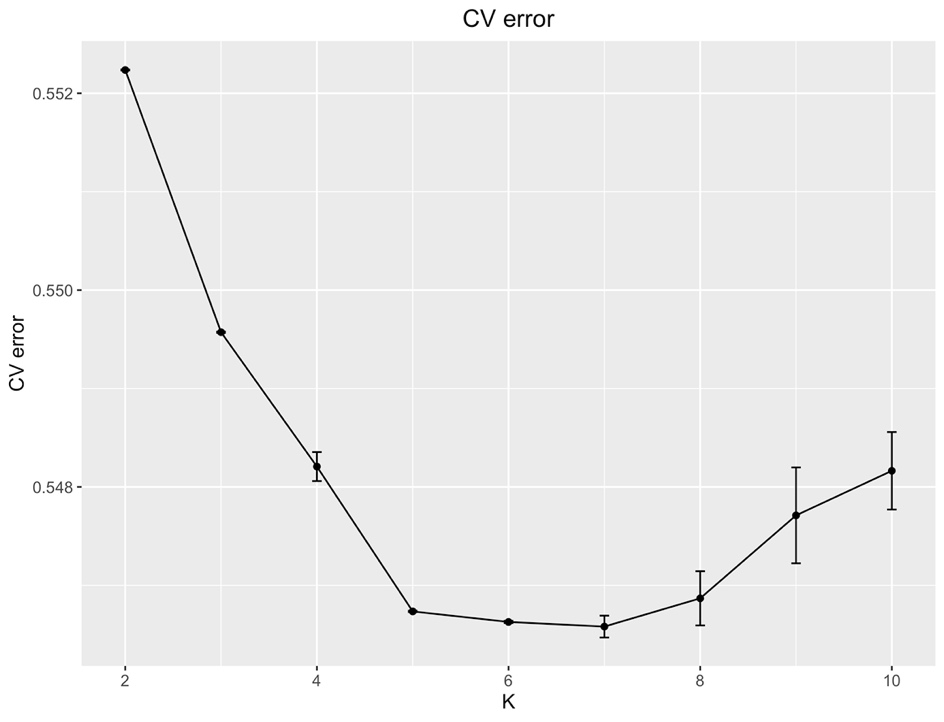
**

**Supplementary Fig. 17 Cross-validation error for ADMIXTURE in Supplementary Fig. 2.**

Cross-validation error for the ADMIXTURE analysis in Supplementary Fig. 2.

| Individual | non-SSLA | SSLA | Individual | non-SSLA | SSLA |
| --- | --- | --- | --- | --- | --- |
| Tunisia_1 | 0.009 | 0.991 | **Morocco_1** | 0.882 | 0.118 |
| Tunisia_2 | 0.891 | 0.109 | **Morocco_2** | 0.895 | 0.105 |
| Tunisia_3 | 0.918 | 0.082 | **Morocco_3** | 0.845 | 0.155 |
| Tunisia_4 | 0.249 | 0.751 | **Morocco_4** | 0.872 | 0.128 |
| Tunisia_5 | 0.016 | 0.984 | **Morocco_5** | 0.775 | 0.225 |
| Tunisia_6 | 0.931 | 0.069 | **Morocco_6** | 0.850 | 0.150 |
| Tunisia_7 | 0.896 | 0.104 | **Morocco_7** | 0.836 | 0.164 |
| Tunisia_8 | 0.881 | 0.119 | **Morocco_8** | 0.929 | 0.071 |
| Tunisia_9 | 0.838 | 0.162 | **Morocco_9** | 0.886 | 0.114 |
| Tunisia_10 | 0.948 | 0.052 | **Morocco_10** | 0.932 | 0.068 |
| Tunisia_11 | 0.936 | 0.064 | **Morocco_11** | 0.908 | 0.092 |
| Tunisia_12 | 0.999 | 0.001 | **Morocco_12** | 0.964 | 0.036 |
| Tunisia_13 | 0.989 | 0.011 | **Morocco_13** | 0.921 | 0.079 |
| Tunisia_14 | 0.949 | 0.051 | **Morocco_14** | 0.930 | 0.070 |
| Tunisia_15 | 0.891 | 0.109 | **Morocco_15** | 0.917 | 0.083 |
| Tunisia_16 | 0.030 | 0.970 | **Morocco_16** | 0.930 | 0.070 |
| Tunisia_17 | 0.845 | 0.155 | **Morocco_17** | 0.890 | 0.110 |
| Tunisia_18 | 0.952 | 0.048 | **Morocco_18** | 0.918 | 0.082 |
| Tunisia_19 | 0.852 | 0.148 | **Morocco_19** | 0.944 | 0.056 |
| Tunisia_20 | 0.927 | 0.073 | **Morocco_20** | 0.866 | 0.134 |
| Tunisia_21 | 0.898 | 0.102 | **Morocco_21** | 0.760 | 0.240 |
| Tunisia_22 | 0.879 | 0.121 | **Morocco_22** | 0.934 | 0.066 |
| Tunisia_23 | 0.856 | 0.144 | **Morocco_23** | 0.945 | 0.055 |
| Tunisia_24 | 0.918 | 0.082 | **Morocco_24** | 0.923 | 0.077 |
| Tunisia_25 | 0.873 | 0.127 | **Morocco_25** | 0.902 | 0.098 |
| Tunisia_26 | 0.860 | 0.140 | **Morocco_26** | 0.922 | 0.078 |
| Tunisia_27 | 0.931 | 0.069 | **Morocco_27** | 0.903 | 0.097 |
| Tunisia_28 | 0.917 | 0.083 | **Morocco_28** | 0.698 | 0.302 |
| Tunisia_29 | 0.985 | 0.015 | **Morocco_29** | 0.913 | 0.087 |
| Tunisia_30 | 0.938 | 0.062 | **Morocco_30** | 0.425 | 0.575 |
| Tunisia_31 | 0.924 | 0.076 | **Morocco_31** | 0.635 | 0.365 |
| Tunisia_32 | 0.832 | 0.168 | **Morocco_32** | 0.874 | 0.126 |
| Tunisia_33 | 0.870 | 0.130 | **Morocco_33** | 0.532 | 0.468 |
| Tunisia_34 | 0.021 | 0.979 | **Morocco_34** | 0.334 | 0.666 |
| Tunisia_35 | 0.730 | 0.270 | **Morocco_35** | 0.411 | 0.589 |
| Tunisia_36 | 0.852 | 0.148 | **Morocco_36** | 0.748 | 0.252 |
| Tunisia_37 | 0.877 | 0.123 | **Morocco_37** | 0.897 | 0.103 |
| Tunisia_38 | 0.895 | 0.105 | **Morocco_38** | 0.312 | 0.688 |
| Tunisia_39 | 0.850 | 0.150 | **Morocco_39** | 0.565 | 0.435 |
| Tunisia_40 | 0.867 | 0.133 | **Morocco_40** | 0.903 | 0.097 |
| Tunisia_41 | 0.881 | 0.119 | **Morocco_41** | 0.892 | 0.108 |
| Tunisia_42 | 0.903 | 0.097 | **Morocco_42** | 0.868 | 0.132 |
| Tunisia_43 | 0.872 | 0.128 | **Morocco_43** | 0.838 | 0.162 |
| Tunisia_44 | 0.916 | 0.084 | **Morocco_44** | 0.932 | 0.068 |
| Tunisia_45 | 0.889 | 0.111 | **Morocco_45** | 0.876 | 0.124 |
| Tunisia_46 | 0.895 | 0.105 |  |  |  |
| Tunisia_47 | 0.888 | 0.112 |  |  |  |
| Tunisia_48 | 0.927 | 0.073 |  |  |  |
| Tunisia_49 | 0.929 | 0.071 |  |  |  |
| Tunisia_50 | 0.902 | 0.098 |  |  |  |
| Tunisia_51 | 0.988 | 0.012 |  |  |  |

**Supplementary Table 4. Local ancestry proportions inferred by RFMix v2**

For each individual, the proportions of non-Sub-Saharan-like and sub-Saharan-like components are given.

**Supplementary Note 1:**

In order to check if the observed closeness between Tunisians and Kenyan populations, and Moroccans and Senegambian populations in the ASPCA, we performed four different t-tests. These compared:

1. The mean distance between the 8 Moroccan haplotypes and Kenyan haplotypes (Luhya, Luo and Bantu from Kenya) to the mean distance between the 10 Tunisian haplotypes and Kenyan haplotypes.
2. The mean distance between the 8 Moroccan haplotypes plus the Tunisian haplotype that clusters with Moroccans in the ASPCA and Kenyan haplotypes (Luhya, Luo and Bantu from Kenya) to the mean distance between the remaining 8 Tunisian haplotypes and Kenyan haplotypes.
3. The mean distance between the 8 Moroccan haplotypes and the Senegambian haplotypes (Gambia and Mandenka) to the mean distance between the 10 Tunisian haplotypes and Senegambian haplotypes.
4. The mean distance between the 8 Moroccan haplotypes plus the Tunisian haplotype that clusters with Moroccans in the ASPCA and the Senegambian haplotypes (Gambia and Mandenka) to the mean distance between the remaining 8 Tunisian haplotypes and Senegambian haplotypes.

All t-tests resulted in a p-value of < 2.2x10^-16^, the lowest value that the analysis can output. These confirmed that, on one hand, independently of in which group the Tunisian closer to Senegambians was placed, Tunisians with >50% of sub-Saharan-like component are significantly closer in the ASPCA to Kenyan haplotypes than to Senegambian haplotypes and are significantly closer to Kenyan haplotypes than Moroccans with >50% of sub-Saharan-like component. On the other hand, Moroccan haplotypes with >50% of sub-Saharan-like component are significantly closer to Senegambian haplotypes than to Kenyan haplotypes, and significantly closer to Senegambian haplotypes than Tunisians.

**Supplementary Note 2:**

While MOSAIC can detect multiple admixture events in a sample (as seen in Fig. 4c), haplotype-based methods like this or the widely used GLOBETROTTER tend to detect the more recent events as stronger signals and can mask older admixture events(Hellenthal et al. 2014; Salter-Townshend and Myers 2019). To address this limitation and explore the possibility of an unseen older admixture event in our data, we performed admixture simulations using AdmixSim (Yang et al. 2020) between a European source (French) and a sub-Saharan source (Yoruba) in a 3:1 proportion, to resemble the results obtained in the studied populations. We simulated one-wave admixture in a range of times in the past starting with the one observed in the observed data (25,50 and 100 generations ago), and a second wave of admixture 25 generations ago (which would coincide with the one inferred in the observed data) after a first wave 50 and 100 generations ago. In this second wave, 2/3 of the resulting population corresponded to the previously admixed sample and 1/3 to Yoruba. We then run local ancestry inference as previously described and assessed the distribution of sub-Saharan-like segments length. Both plots visualization (Supplementary Fig. 11,12) and Kolmogorov-Smirnov tests (Supplementary Table 3) confirm that the distribution observed in the whole-population observed data corresponds to a one-wave admixture, and while we cannot fully reject the existence of older admixture events, it seems clear that current North African’s sub-Saharan-like ancestry is linked to the recent admixture events we have detected.
